# Synthesis and in vivo evaluation of a bifunctional, glutamic acid derived chelator for the ^203/212^Pb theranostic pair

**DOI:** 10.1186/s41181-026-00473-9

**Published:** 2026-07-04

**Authors:** Jarred Michael Scaffidi-Muta, Kwong Ching Li, Didier Boucher, Thomas Kryza, William Tieu, Andrew David Abell

**Affiliations:** 1 School of Physics, Chemistry & Earth Sciences, Adelaide University, Adelaide, South Australia Australia; 2 AdvanCell Pty Ltd., Richlands, QLD Australia

**Keywords:** Lead-212, Lead-203, Bifunctional chelator, MC1R, Melanoma, Radiopharmaceutical

## Abstract

**Background:**

The biodistributions of radiometalated peptides and small molecules are greatly influenced by the charge conferred by the metal-chelator complex. Careful fine-tuning of this charge thus represents an attractive method to optimise pharmacokinetic properties. For this to be an effective strategy, numerous suitable chelators must be available for a given radiometal; each possessing a different net charge. Herein we report the synthesis of 2-(4,7,10-tris(2-amino-2-oxoethyl)-1,4,7,10-tetraazacyclododecan-1-yl)pentanedioic acid (DOTAMGA), a bifunctional chelator for the ^203/212^Pb theranostic pair containing a single negative charge, previously unattainable with reported radiolead chelators. DOTAMGA was incorporated into a radiopharmaceutical targeting the melanocortin 1 receptor (MC1R), a receptor highly expressed by melanomas, and evaluated in vivo against the commonly used radiolead chelator 1,4,7,10-tetraazacyclododecane-1,4,7,10-tetraacetic amide (TCMC).

**Results:**

DOTAMGA was synthesised and conjugated to the MC1R targeting peptide MC1RL for subsequent investigation alongside TCMC-MC1RL. DOTAMGA-MC1RL and TCMC-MC1RL exhibited comparable affinity for MC1R in a series of competition binding assays with MC1R expressing cells. DOTAMGA-MC1RL was effectively labelled with ^212^Pb and ^203^Pb under standard radiolabelling conditions, and UV–Vis experiments demonstrated more rapid Pb^2+^ complexation than TCMC-MC1RL. Both ^212^Pb-labelled compounds remained > 90% intact after 24 h incubation in human serum. In B16F0 melanoma bearing C57BL/6 mice, DOTAMGA-MC1RL exhibited reduced hepatic uptake compared to TCMC-MC1RL, though a reduction in tumour uptake and resulting tumour to organ uptake ratios were also observed.

**Conclusions:**

DOTAMGA represents a promising tool to optimise the pharmacokinetic properties of ^203/212^Pb radiopharmaceuticals, and is a useful addition to existing radiolead chelators on account of its unique charge.

**Supplementary Information:**

The online version contains supplementary material available at https://doi.org/10.1186/s41181-026-00473-9.

## Background

Targeted radionuclide therapy (TRT) is emerging as a safe and effective alternative to conventional radiotherapies (Sgouros et al. 2020; Stokke et al. 2022). In TRT, radionuclides which emit cytotoxic, but short ranged radiation are selectively delivered to tumours via conjugation to a tumour targeting vector. In this way, a therapeutic dose of radiation can be administered whilst minimising damage to surrounding healthy tissues. Radionuclides which emit α particles are particularly well-suited for use in TRT, on account of their short range in tissue (< 100 μm) and exquisite cytotoxicity (Guerra Liberal et al. 2020 ; Group 2018). For this reason, ^212^Pb has attracted considerable interest for use in TRT through its α-emitting ^212^Bi daughter (Kokov et al. 2022; Scaffidi-Muta and Abell 2025). Compared to other α-emitters, ^212^Pb is easily produced, exhibits favourable chemistry, and has a short 10.6 h half-life. ^212^Pb is also elementally matched to the diagnostic nuclide ^203^Pb, a γ-emitter readily imaged by single-photon emission computed tomography (SPECT). ^203^Pb and ^212^Pb have thus been dubbed a ‘theranostic pair’, allowing for image-guided cancer therapies (McNeil et al. 2023; Nelson et al. 2023).

Since Pb^2+^ does not inherently accumulate in cancerous tissues, tumour localisation is facilitated using a targeting vector which selectively binds to cancer cells. This is typically achieved using a bifunctional chelator, which contains both a metal binding moiety, and a reactive functional group to enable covalent attachment to the targeting vector. Bifunctional derivatives of 1,4,7,10-tetraazacyclododecane-1,4,7,10-tetraacetic acid (DOTA) and its tetra-amide analogue 1,4,7,10-tetraazacyclododecane-1,4,7,10-tetraacetic amide (TCMC a.k.a. DOTAM) are usually employed for radiolead chelation, as their resulting Pb^2+^ complexes are thermodynamically stable, and both kinetically and biologically inert (Grieve and Paterson 2022; Pippin et al. 1995). TCMC has emerged as the standard chelator for ^203/212^Pb over DOTA, on account of its greater acidic stability, and more facile radiolabelling (Chappell et al. 2000; Scaffidi-Muta and Abell 2025).

**Fig. 1:**
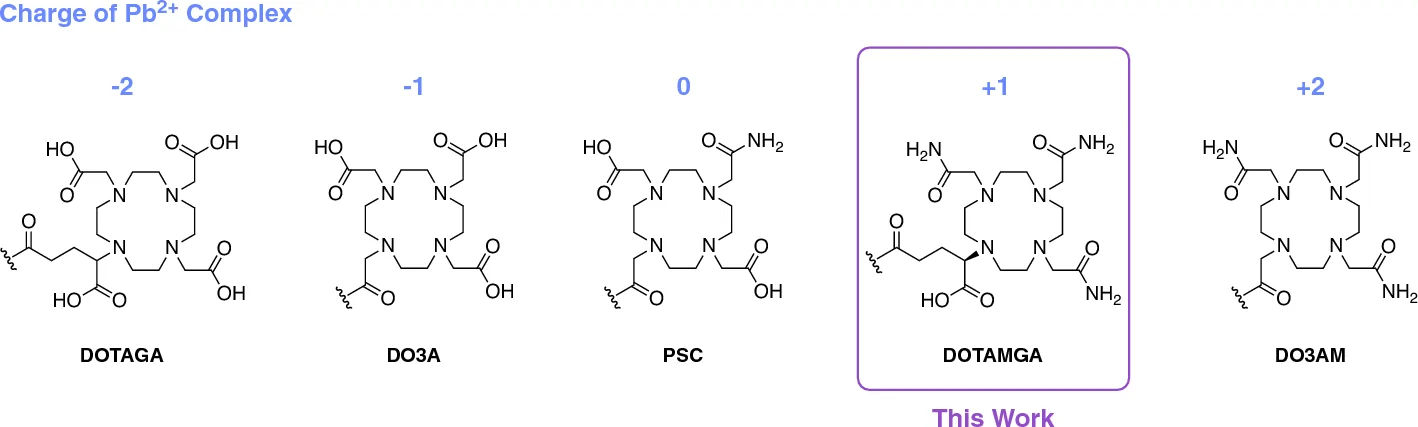
Bifunctional chelators employed with peptidic and small molecule ^203/212^Pb radiopharmaceuticals. Net charges of the resulting Pb^2+^ complexes under physiological conditions are indicated above each structure in blue

With peptidic and small molecule targeting vectors, the bifunctional chelator employed can greatly impact the pharmacokinetic parameters of the resulting radiopharmaceutical (Bauer et al. 2024; Lee et al. 2024; Li et al. 2023; Saidi et al. 2025). As the chelator is generally not expected to participate in target binding, it thus represents an attractive site for chemical modification to improve the biological properties of a radiopharmaceutical without compromising its targeting affinity. Net charge specifically can have an outsized effect on a radiopharmaceutical’s biodistribution and route of elimination (Akizawa et al. 2001; Bauer et al. 2024; Raheem et al. 2023). While decreasing net charge tends to decrease hepatic and renal uptakes, the trend is not universal, and the optimal charge should be experimentally determined in each instance. To this end, numerous bifunctional chelators have been employed to fine-tune the charge of ^203/212^Pb radiopharmaceuticals, as depicted in Fig. 1. (4,7,10-tris(2-amino-2-oxoethyl)-1,4,7,10-tetraazacyclododecan-1-yl)acetic acid (DO3AM) is the bifunctional TCMC derivative most commonly used with peptidic radiopharmaceuticals, which creates a + 2 charged Pb^2+^ complex due to its lack of formal charge (Jurek et al. 2018). On the other hand, the DOTA derivatives DO3A and 2-(4,7,10-tris(carboxymethyl)-1,4,7,10-tetraazacyclododecan-1-yl)pentanedioic acid (DOTAGA) have been employed to respectively produce -1 and -2 charged Pb^2+^ complexes (Bauer et al. 2024; Saidi et al. 2025). Pb specific chelator (PSC) was developed to explore charges between these extremes, bearing just two carboxylates to confer a neutral net charge to its resulting Pb^2+^ complex (Li et al. 2023). However, PSC exhibits slower and less efficient radiolabelling than either DO3AM or DO3A (Saidi et al. 2025).

For this study, we aimed to develop DOTAMGA as an addition to the repertoire of reported radiolead chelators. With its single carboxylate, its Pb^2+^ complex has a net charge of + 1, previously unattainable with existing bifunctional chelators. Moreover, unlike PSC, DOTAMGA has all four of its pedant arms available for radiometal chelation, and thus we also anticipated it could maintain the rapid and robust radiolabelling of DO3AM. After synthesis, DOTAMGA was conjugated to the melanocortin 1 receptor (MC1R) targeted peptide MC1RL for further evaluation as a melanoma therapy (Barkey et al. 2011; Khushalani et al. 2024; López et al. 2007; Siegrist et al. 1989; Tafreshi et al. 2019).

## Results

### Synthesis

**Scheme 1:**
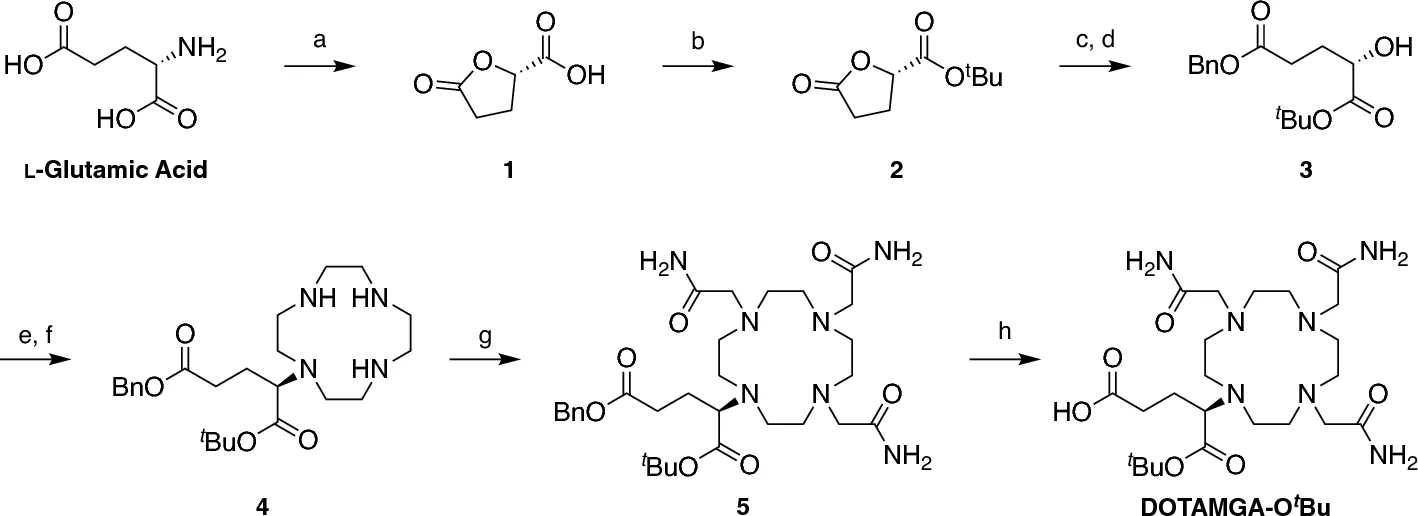
Synthesis of DOTAMGA-O^*t*^Bu. Reagents and conditions: *a*) NaNO_2_, HCl, H_2_O, 0 °C, 16 h, 77%. *b*) DIC, DMAP, ^*t*^BuOH, DCM, r.t., 16 h, 86%. *c*) KOH, H_2_O, THF, 40 °C, 2 h. *d*) BnBr, DMF, r.t., 16 h, 78% (2 steps). *e*) MsCl, TEA, DCM, r.t, 30 min. *f*) cyclen, K_2_CO_3_, MeCN, 50 °C, 20 h, 74% (2 steps). *g*) 2-bromoacetamide, K_2_CO_3_, MeCN, 50 °C, 20 h, 66%. *h*) 10% Pd/C, NH_4_HCO_2_, MeOH, reflux, 4 h, quant

DOTAMGA was synthesised as a *tert*-butyl ester, such that it was compatible with typical Fmoc-based solid phase peptide synthesis techniques (Chan and White 1999). The synthesis was analogous to that previously reported for *R*-DOTAGA-(O^*t*^Bu)_4_ (Levy et al. 2009), and is depicted in Scheme 1. Starting from l-glutamic acid, diazotisation facilitated the stereoselective esterification to give lactone **1** in a 77% yield. This was converted to *tert*-butyl ester **2** in an 86% yield via an *N*,*N*′-diisopropylcarbodiimide and 4-dimethylaminopyridine mediated Steglich esterification with *tert*-butanol. The lactone was hydrolysed, and the resulting carboxylate salt alkylated upon treatment with benzyl bromide to give alcohol **3** (78% yield for two steps). Alcohol **3** was converted to its mesylate ester, which was subsequently used to alkylate cyclen (1,4,7,10-tetraazacyclododecane) in the presence of potassium carbonate to give monoalkylated cyclen **4**. To favour formation of the monoalkylated product, the mesylate ester of **3** was added dropwise to an excess of cyclen, which was subsequently removed by thoroughly washing with water during the work up. Characterisation data for compounds **1**–**4** was consistent with the literature (Eisenwiener et al. 2000; Levy et al. 2009). Fully alkylated **5** could then be prepared in a 66% yield using an excess of 2-bromoacetamide and potassium carbonate. Finally, the benzyl ester was quantitatively cleaved via palladium catalysed hydrogenation to give DOTAMGA-O^*t*^Bu.

**Fig. 2:**
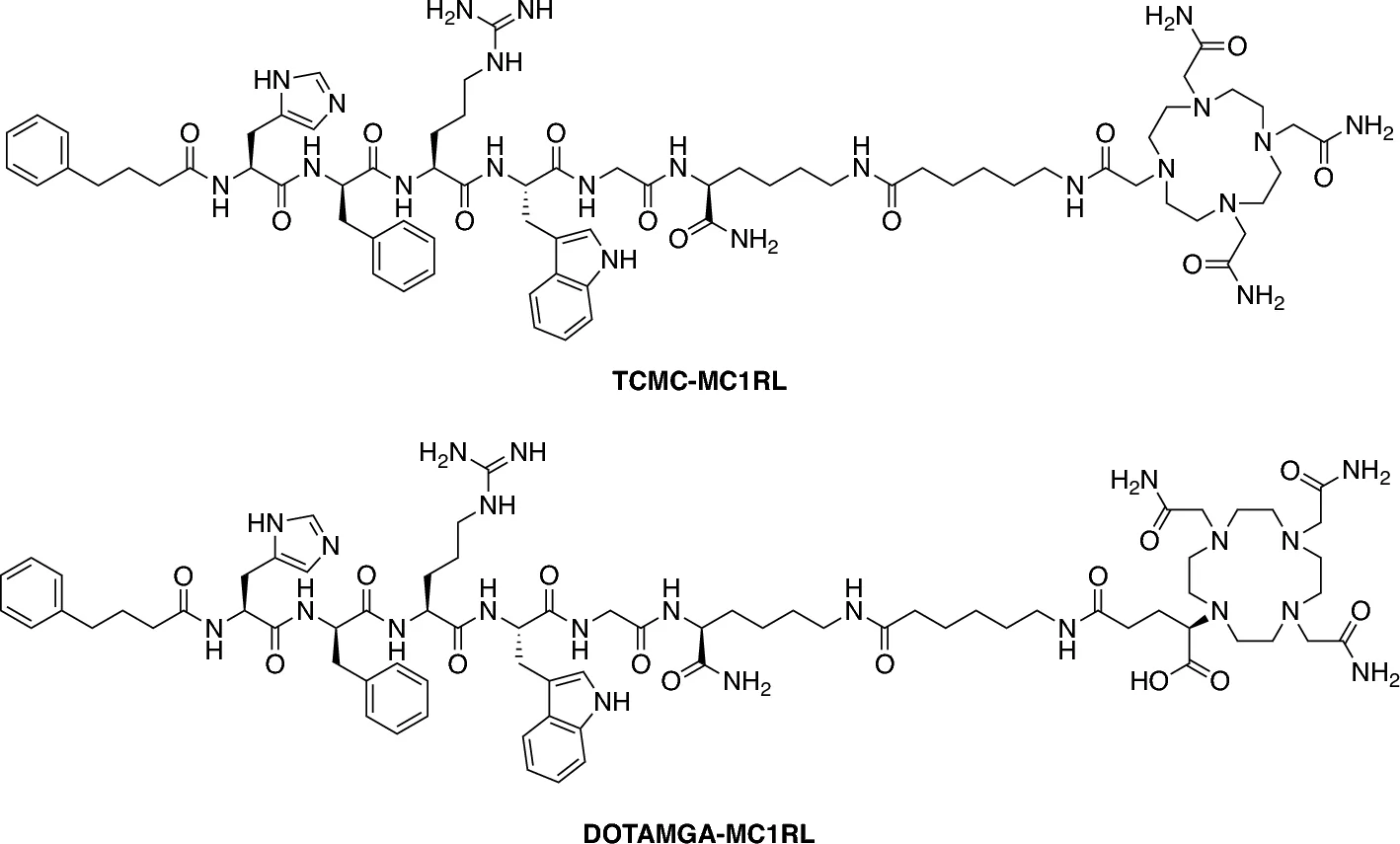
MC1R targeted peptides synthesised in this study

TCMC-MC1RL and DOTAMGA-MC1RL (Fig. 2) were then synthesised according to standard solid phase peptide synthesis procedures (Chan and White 1999). After synthesis of MC1RL, the corresponding chelator was coupled to the aminohexanoic acid amine on resin. DOTAMGA-O^*t*^Bu could be readily conjugated to the peptide using HCTU, and its *tert*-butyl ester subsequently deprotected under standard peptide cleavage and global deprotection conditions to afford DOTAMGA-MC1RL.

### MC1R binding affinity

MC1R binding affinity was expressed as half maximal inhibitory concentration values (IC_50_) derived from cellular competition binding assays against [^125^I]NDP-MSH using melanoma cells (SK-MEL-13) overexpressing MC1R (Table 1). Selectivity was likewise assessed against MC4R, a closely related melanocortin receptor, by performing analogous competition binding assays using MC4R overexpressing SK-MEL-13 cells. For comparison, MC1R affinity and selectivity of the potent melanocortin receptor ligand NDP-MSH was also assessed (Sawyer et al. 1980). Both TCMC-MC1RL and DOTAMGA-MC1RL exhibited high affinity for MC1R, with comparable nanomolar IC_50_ values for MC1R which were each lower than that of NDP-MSH. Both compounds were selective for MC1R, with each exhibiting IC_50_ values for MC4R greater than those for MC1R.

**Table 1:** MC1R affinity and selectivity

Compound	MC1R IC_50_ (nM) ^a^	MC4R IC_50_ (nM)^a^
TCMC-MC1RL	2.1 ± 0.23	28 ± 3.9
DOTAMGA-MC1RL	2.4 ± 0.43	100 ± 10
NDP-MSH	4.3 ± 0.42	130 ± 28

^a^ Reported as mean ± standard error (*n* = 3)

### Formation kinetics

**Fig. 3:**
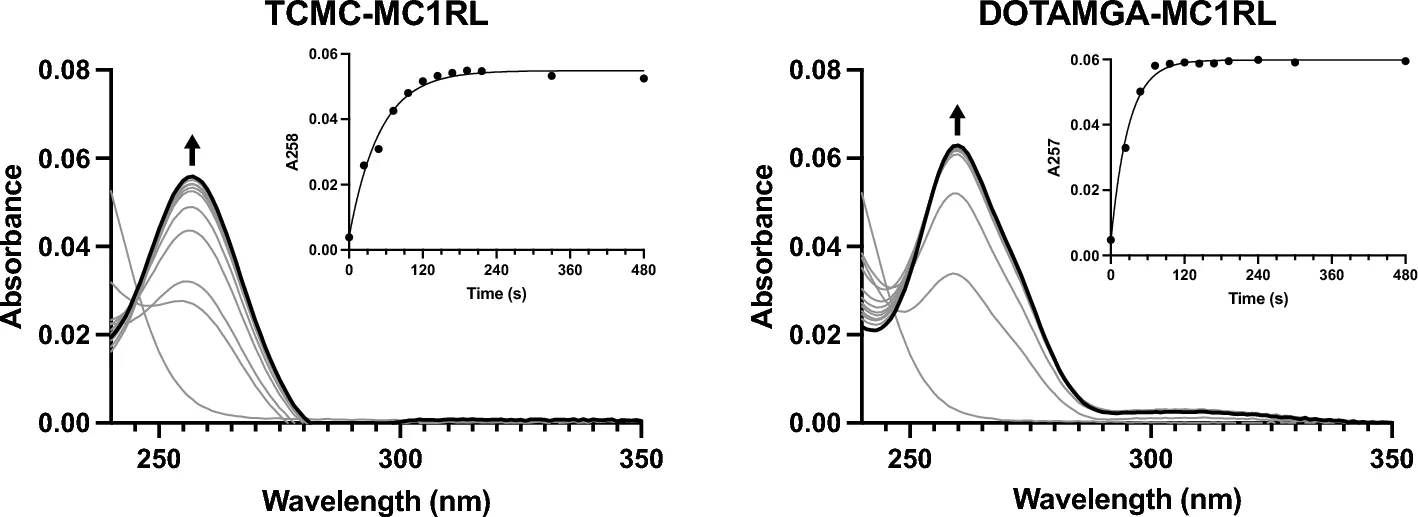
UV–Vis spectra and peak absorbance (inset) over time of the complexation between Pb^2+^ and MC1R targeted ligands. Reactions performed at pH = 5.5 in 0.1 M sodium acetate with 0.01 mM PbCl_2_ and 0.02 mM ligands at ambient temperature

Formation kinetics of the Pb-labelled peptides were investigated using UV–vis absorption spectrophotometry. Reactions were performed at ambient temperature in 0.1 M sodium acetate pH = 5.5 to emulate typical radiolabelling conditions, with 0.01 mM and 0.02 mM respectively of PbCl_2_ and ligand. Pb-peptide complexes could be detected by their 257–258 nm absorption peaks, attributable to the 6s^2^ → 6sp transition of Pb^2+^ (Kumar et al. 1989; Pippin et al. 1995; Tosato et al. 2022). As shown in Fig. 3, DOTAMGA-MC1RL exhibited the greatest rate of Pb^2+^ complexation, reaching equilibrium by 72 s after the addition of PbCl_2_, compared to the approximately 170 s required for TCMC-MC1RL.

### Radiolabelling

**Fig. 4:**
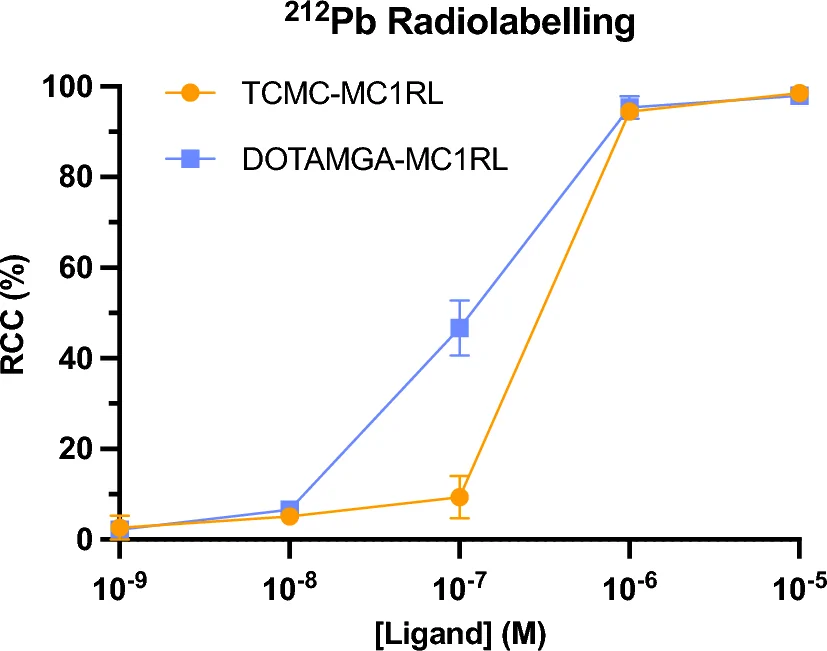
Radiolabelling of TCMC-MC1RL and DOTAMGA-MC1RL with 0.5 MBq ^212^PbCl_2_ in 0.1 M NaOAc pH = 5.5 after heating at 30 ºC for 20 min. Results reported as mean ± standard deviation (n = 3)

A series of radiolabelling experiments were performed to compare radiolabelling efficiency of the two chelators. The synthesised peptides were radiolabelled with 0.5 MBq ^212^Pb in 0.1 M sodium acetate buffer at pH = 5.5, and radiochemical conversions (RCC) were determined by radio-TLC analysis of the reaction mixture after heating at 30 ºC for 20 min. Both compounds achieved RCCs above 90% at concentrations of 10^–5^ M and 10^–6^ M, whist RCCs at 10^–8^ M and 10^–9^ M were below 10% (Fig. 4). At 10^–7^ M, TCMC-MC1RL exhibited an RCC of 9.3 ± 4.6%, while that for DOTAMGA-MC1RL was significantly greater at 47 ± 6.1% (*p* = 0.001, *n* = 3).

TCMC-MC1RL and DOTAMGA-MC1RL were analogously radiolabelled with ^212^Pb and ^203^Pb for use in subsequent in vitro and in vivo experiments. While RCCs typically exceeded 99% for both nuclides, ^203^Pb radiolabelling for the in vivo experiments was incomplete after heating at 30 °C for 20 min (Table S2). We hypothesised this to be caused by metal ion contamination of the ^203^PbCl_2_ solution given the success of the ^212^Pb radiolabelling, and the ^203^Pb labelling for the serum stability studies. While a radiochemical conversion of > 99% was achieved for DOTAMGA-MC1RL after heating at 50 °C for 20 min, these conditions produced a conversion of just 41% for TCMC-MC1RL. After an additional 30 min at 50 °C, a conversion of 94% was achieved for TCMC-MC1RL. The ^203^Pb radiolabelled peptides were then formulated with 0.25 mM diethylenetriaminepentaacetic (DTPA) and 0.25 M sodium ascorbate for the biodistribution experiments, the latter of which effectively mitigated radiolysis of the peptide (Figure S1).

### Serum stability

The kinetic stabilities of the ^212^Pb-chelator complexes were investigated by incubation in human serum for 48 h at 37 °C, followed by radio-TLC analysis (Table 2). Minimal release of ^212^Pb from either compound was observed under these conditions, with over 96% remaining bound by end of the study period in either case. The Pb-DOTAMGA complex thus appeared comparatively inert as the analogous Pb-TCMC complex.

**Table 2:** In vitro stability of ^212^Pb-labelled peptides in human serum at 37 °C

	^212^Pb Bound (%)			
Compound	0 h	5 h	24 h	48 h
TCMC-MC1RL	98.5	98.3	97.3	97.1
DOTAMGA-MC1RL	97.3	97.2	96.8	96.6

**Fig. 5:**
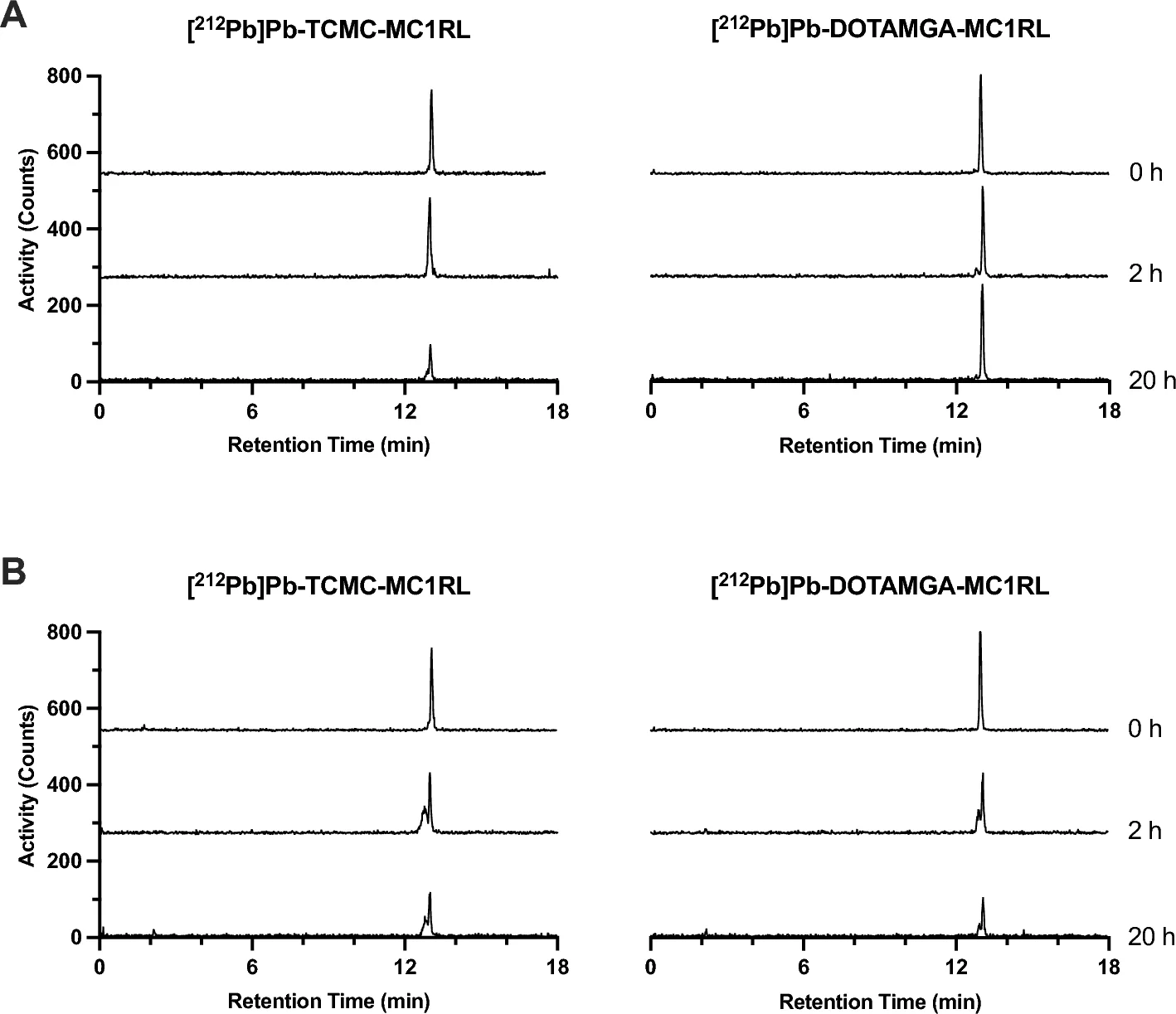
Stability of the ^203^Pb-labelled peptides after incubation in **A** pH = 7.4 PBS or **B** human serum at 37 ºC

The stability of the ^203^Pb-radiolabelled peptides was also assessed by radio-HPLC following incubation in either 0.1 M phosphate buffered saline (PBS) pH = 7.4, or human serum (Fig. 5). While [^203^Pb]Pb-DOTAMGA-MC1RL retained a radiochemical purity above 90% after 20 h incubation in PBS, [^203^Pb]Pb-TCMC-MC1RL remained only 75% intact under the same conditions, evidence of radiolytic degradation. Both compounds were similarly degraded by the serum, with each having a radiochemical purity of around 50% after 20 h incubation.

### Lipophilicity

The lipophilicities of the ^212^Pb-labelled compounds were evaluated as the octanol–water distribution coefficients (log D_7.4_) using a miniaturised shake flask assay and expressed as mean ± standard deviation. The calculated log D_7.4_ values for [^212^Pb]Pb-TCMC-MC1RL and [^212^Pb]Pb-DOTAMGA were − 1.8 ± 0.07 and − 2.1 ± 0.07 respectively. [^212^Pb]Pb-DOTAMGA was thus more hydrophilic than [^212^Pb]Pb-TCMC-MC1RL (*p* = 0.009, *n* = 3).

### In Vivo* biodistribution*

**Fig. 6:**
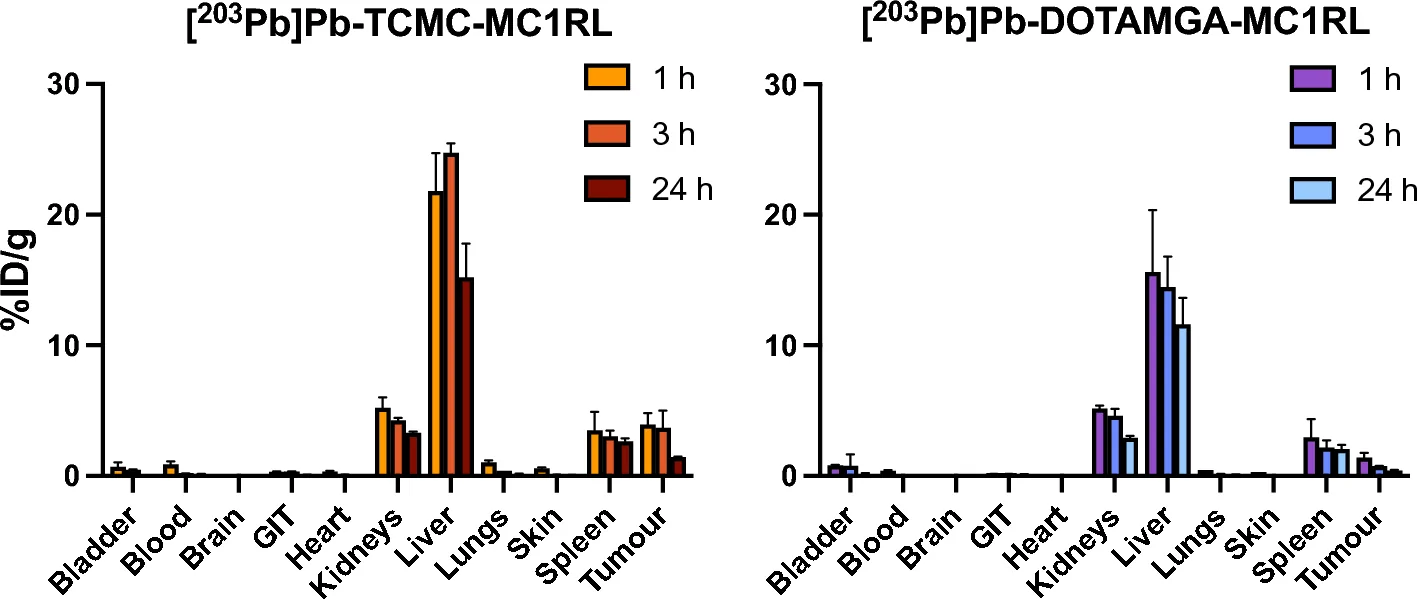
In vivo biodistribution and tumour to organ uptake ratios of ^203^Pb-labelled compounds in female C57BL/6 mice bearing B16F0 tumours in the right flank. 99 kBq of each compound was administered on average, radiolabelled at a specific activity of 2.0 MBq/μg. Results are presented as mean %ID/g ± standard deviation (*n* = 2 for TCMC-MC1RL at 1 h, *n* = 3 for all other data). GIT: Gastrointestinal tract

Biodistribution of the ^203^Pb labelled peptides was determined in female C57BL/6 mice bearing B16F0 murine melanoma tumours (Fig. 6). Both compounds primarily accumulated in the kidneys, liver, spleen, and tumour. While maximal uptakes were observed in all organs 1 h post injection for [^203^Pb]Pb-DOTAMGA-MC1RL, hepatic uptake of [^203^Pb]Pb-TCMC-MC1RL was at its greatest 3 h post injection. Renal and splenic uptakes were comparable between the two compounds (*p* > 0.05), though [^203^Pb]Pb-TCMC-MC1RL exhibited far greater hepatic uptake than [^203^Pb]Pb-DOTAMGA-MC1RL (25 ± 0.73%ID/g vs 14 ± 2.3%ID/g 3 h post injection, *p* = 0.005, *n* = 3). Tumour uptake of [^203^Pb]Pb-TCMC-MC1RL was also much greater compared to [^203^Pb]Pb-DOTAMGA-MC1RL (3.7 ± 1.3%ID/g vs 0.73 ± 0.018%ID/g 3 h post injection respectively, *p* = 0.02, *n* = 3). As a surrogate for absorbed dose, the integral of the organ uptakes over time (area under curve, AUC) were also determined (Table 3) (Saidi et al. 2025). While AUC values for the kidneys and spleen were equivalent for both compounds, those for the liver and tumour were again higher with [^203^Pb]Pb-TCMC-MC1RL (*p* = 0.02 and 0.03 respectively, *n* = 3). [^203^Pb]Pb-TCMC-MC1RL also exhibited greater retention in the blood than [^203^Pb]Pb-DOTAMGA-MC1RL (*p* = 0.001, *n* = 3).

**Table 3:** AUC of ^203^Pb-labelled, MC1R targeted peptides

	AUC ^a^				
Compound	Tumour	Kidneys	Liver	Spleen	Blood
TCMC-MC1RL	61 ± 14	89 ± 2.6	470 ± 29	66 ± 5.4	4.7 ± 0.34
DOTAMGA-MC1RL	14 ± 0.67	89 ± 6.0	300 ± 33	49 ± 6.9	1.4 ± 0.18

^a^ Area of polygon defined by %ID/g organ uptake values at 1, 3, and 24 h post injection ± standard error

**Fig. 7:**
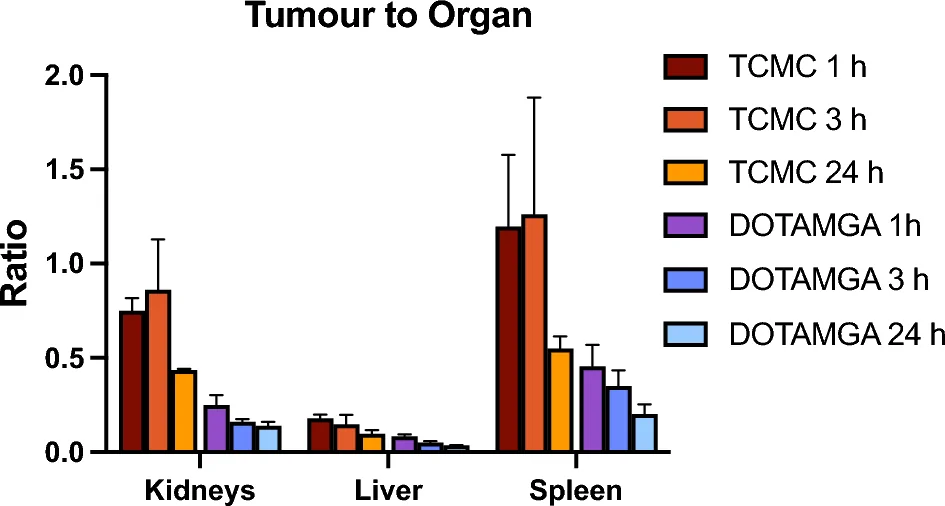
Tumour to normal organ uptake ratios for ^212^Pb-labelled MC1R targeted peptides TCMC-MC1RL and DOTAMGA-MC1RL. Ratios were calculated by dividing the tumour %ID/g values by the respective organ %ID/g values at each time point. Results are presented as mean ± standard deviation (*n* = 2 to 3)

To compare the tumoural specificity, the ratio of tumour to normal organ uptake was calculated at each time point for each compound (Fig. 7). Ratios greater than one were indicative of preferential uptake in the tumour than the respective normal organ, and vice versa. Mean tumour to kidney and tumour to spleen uptake ratios were less than 1 at each time point for both compounds. Tumour to spleen uptake ratios were also below 1 for [^203^Pb]Pb-DOTAMGA-MC1RL throughout the experiment, and for [^203^Pb]Pb-TCMC-MC1RL at 24 h post injection. Although [^203^Pb]Pb-DOTAMGA-MC1RL exhibited reduced accumulation in the liver, its lower tumour uptake resulted in inferior tumour to liver uptake ratios to those of [^203^Pb]Pb-TCMC-MC1RL (*p* < 0.05, *n* = 3). Tumour to kidney uptake ratios of [^203^Pb]Pb-DOTAMGA-MC1RL were also significantly less than those for [^203^Pb]Pb-TCMC-MC1RL at all time points (*p* < 0.05, *n* = 3).

## Discussion

Radiometal based TRT generally necessitates bifunctional chelating agents to sequester therapeutically relevant radionuclides and conjugate them to appropriate targeting vectors. Such chelators must be capable of forming kinetically and biologically inert radiometal complexes, ideally rapidly and under mild conditions. This chelator can itself influence the pharmacokinetic profile of the resulting radiopharmaceutical, particularly when employing small molecule or peptidic targeting vectors. As it is typically not expected to participate in target binding, altering the chelator employed in a given radiopharmaceutical thus represents an attractive strategy to optimise pharmacokinetic properties. For this to be effective, numerous viable chelators must be available for a given radiometal, each with differing physical and chemical properties.

Research on radiolead based TRT is currently dominated by the use of TCMC on account of its rapid and stable complexation with Pb^2+^ (Kokov et al. 2022; Scaffidi-Muta and Abell 2025). However, alternative chelators are beginning to be more widely adopted as tools to alter biological properties (Bauer et al. 2024; Lee et al. 2024; Li et al. 2023; McNeil et al. 2024; Saidi et al. 2025). Chief among these are DOTA and PSC, each decreasing the net charge of their resulting Pb^2+^ complexes on account of their carboxylate pendent arms. Two limitations to this strategy are however apparent. While PSC decreases net charge by 2 relative to TCMC, and DOTA by 3 or 4 depending on the bifunctional derivative used, no such chelator is available to decrease the charge by just 1. Additionally, both PSC and DOTA require harsher conditions than TCMC for efficient radiolabelling (Saidi et al. 2025). DOTAMGA was thus designed to address these limitations. Possessing a single carboxylate pendant arm, its net charge is reduced by 1 compared to TCMC. Additionally, while PSC and TCMC are typically conjugated to peptidic and small molecule targeting vectors by amidation of their pendent arms, DOTAMGA is conjugated such that all four of its pendant arms remain free to participate in Pb^2+^ complexation.

The *tert*-butyl protected DOTAMGA-O^*t*^Bu was synthesised analogously to DOTAGA-(O^*t*^Bu)_4_ (Levy et al. 2009), and could be readily conjugated to a peptidic MC1R targeting vector on-resin following standard solid phase peptide synthesis procedures. Under conditions typical of ^203/212^Pb radiolabelling, kinetic studies indicated that the DOTAMGA conjugated peptide exhibited more rapid Pb^2+^ complexation than the analogous TCMC conjugate. DOTAMGA-MC1RL likewise had a greater ^212^Pb radiolabelling efficiency than TCMC-MC1RL at 10^–7^ M of ligand. Since TCMC was conjugated to MC1RL via one of its pendant arms, Pb^2+^ complexation may have be accordingly impaired compared to DOTAMGA. Importantly, complexed ^212^Pb remained stably bound to both ligands when incubated in human serum, suggesting the Pb-DOTAMGA complex shares the kinetic inertness exhibited by Pb-TCMC. MC1R binding assays demonstrated only minimal impact of the chelator on receptor binding, with both compounds exhibiting comparable affinity and selectivity to MC1R. The chelator thus appears to be a viable site for further chemical modification in a future series of MC1R targeted compounds. As both compounds in this study exhibited considerable degradation in serum, future analogues may also benefit from modifications to improve enzymatic stability (Cooper et al. 2021).

As has been observed with other peptidic MC1R targeted radiopharmaceuticals, the kidneys, liver, spleen, and tumour represented the main accumulating organs for both compounds (Miao et al. 2005; Shi and Cheng 2022; Tafreshi et al. 2019; Tichacek et al. 2020; Zhang et al. 2017). Decreasing a radiopharmaceutical’s net charge is generally thought to inhibit renal retention by disrupting the electrostatic interactions between it and the negatively charged brush boarder and proximal tubule surfaces (Akizawa et al. 2008). Such a trend was not however observed in the present study, with both compounds exhibiting comparable renal uptake despite their differing charges. A greater reduction in charge may be necessary to observe this effect, or kidney retention may alternatively be impacted by other factors such as charge distribution (Akizawa et al. 2008). Hepatic uptake was far more sensitive to charge, with the mean uptake of [^203^Pb]Pb-DOTAMGA-MC1RL almost half that of [^203^Pb]Pb-TCMC-MC1RL. This trend has been reported for other peptide and protein targeted radiopharmaceuticals and may be attributable to increased hydrophilicity which increases the rate of elimination (Chen et al. 2000; Froidevaux et al. 2005; Hosseinimehr et al. 2012; Rinne et al. 2019). The comparatively faster elimination of [^203^Pb]Pb-DOTAMGA-MC1RL than [^203^Pb]Pb-TCMC-MC1RL appears to be supported by the lower blood retention exhibited by the former compound.

The concomitant reduction in tumour uptake in this study upon the addition of a charged functional group has also been observed with other MC1R targeted radiopharmaceuticals (Chen et al. 2000; Rinne et al. 2019) While this may also result from the more rapid pharmacokinetic profile of [^203^Pb]Pb-DOTAMGA-MC1RL compared to [^203^Pb]Pb-TCMC-MC1RL, the increased hydrophilicity and charge of the former compound may reduce tumour uptake by inhibiting cellular internalisation. MC1R agonist activity is greatly affected by the confirmation of the ligand (Cai et al. 2005), and thus the DOTAMGA chelator may alter peptide confirmation such that agonism is decreased relative to TCMC-MC1RL despite their comparable MC1R affinities. In vivo blocking studies would help confirm that the disparity in tumour uptake observed was a result of MC1R mediated targeting, rather than differences in non-specific uptake.

The potential efficacy of a TRT is determined not solely by the tumour absorbed dose, but by the relationship between this dose and that absorbed by normal tissues. Accordingly, while [^203^Pb]Pb-DOTAMGA-MC1RL exhibited reduced hepatic retention relative to [^203^Pb]Pb-TCMC-MC1RL, the associated reduction in tumour uptake conferred less favourable tumour to organ uptake ratios. In this instance, the use of DOTAMGA in place of TCMC thus appeared reduce the therapeutic viability of the resulting compound. This profound impact on biodistribution conferred by the addition of a single negative charge does however demonstrate the importance of optimising a radiopharmaceutical’s structure with respect to charge. DOTAMGA has recently been successfully employed in the prostate specific membrane antigen targeted compound [^212^Pb]Pb-ADVC001, currently being evaluated for the treatment of metastatic castration-resistant prostate cancer in a phase I/II clinical trial (Liu et al. 2025). As an addition to the repertoire of existing radiolead chelators, we thus anticipate DOTAMGA to be a valuable tool in optimising the pharmacokinetic properties of other ^203/212^Pb based therapies.

## Conclusion

A bifunctional chelator for the ^203/212^Pb theranostic pair was synthesised and conjugated to the peptidic MC1R targeting vector MC1RL. DOTAMGA-MC1RL maintained a high affinity for the MC1R receptor, exhibited more rapid Pb^2+^ complexation than TCMC-MC1RL, and could be efficiently radiolabelled under mild conditions. While [^203^Pb]Pb-DOTAMGA-MC1RL exhibited reduced hepatic uptake than [^203^Pb]Pb-TCMC-MC1RL, a concomitant reduction in tumour uptake conferred inferior tumour to normal organ uptake ratios. Despite this, this study demonstrates the importance of charge in optimising biodistribution, and we anticipate DOTAMGA to be a useful tool in optimising other radiolead based radiopharmaceuticals for TRT.

## Methods

### General

Reagents and solvents were purchased from Chem-Impex, ChemSupply, Macrocyclics, Revvity, Sigma-Aldrich, and Thermo Fisher Scientific, and used without further purification. [^203^Pb]PbCl_2_ in 0.05 M HCl was purchased from The University of Alabama at Birmingham. [^212^Pb]PbCl_2_ in 0.05 M HCl was obtained from AdvanCell. Optical rotations were determined using an Anton Paar MCP 100 polarimeter and are reported as 10^−1^ deg cm^2^ g^−1^. NMR spectroscopy was performed using a Bruker 600 MHz Ascend NMR spectrometer, or an Agilent 500 MHz DD2 NMR spectrometer. ^1^H and ^13^C NMR spectra were referenced to tetramethylsilane and *J* values are given in Hz. ^1^H and ^13^C NMR spectra for compounds **1** to **5** and DOTAMGA-O^*t*^Bu are reported in Figures S2 to S7 of the supplementary information. High resolution mass spectrometry was performed using an Agilent 6230 HRMS TOF-LCMS system. HPLC purification was achieved using an Agilent 1260 Infinity II preparative HPLC system equipped with a 150 × 21.2 mm Agilent InfinityLab ZORBAX Eclipse Plus C18 column. UV–Vis absorption spectra were acquired using an Agilent Cary 5000 UV–Vis-NIR spectrophotometer.

### Chelator Synthesis

#### (2S)-5-Oxooxolane-2-carboxylic acid (1)

l-Glutamic acid (10.1 g, 59.9 mmol) was dissolved in a solution of water (50 mL) and concentrated hydrochloric acid (15.0 mL, 175 mmol), and cooled in an ice bath with stirring. A solution of sodium nitrite (6.28 g, 91.0 mmol) in water (25 mL) was added dropwise over 20 min, then reaction mixture was left to stir for 6 h at ambient temperature. Solvent was removed *in vacuo* at 30 °C, and the residue dissolved in ethyl acetate (200 mL). The solution was dried over anhydrous magnesium sulphate, filtered, and solvent removed *in vacuo* to afford lactone **1** as a pale yellow solid (5.98 g, 77%). [α]^25^_D_ =  + 12.1 (*c* 1.0, EtOH). ^1^H NMR *(*600 MHz, DMSO-*d*_*6*_): δ 13.12 (bs, 1H), 5.01 – 4.93 (m, 1H), 2.56 – 2.42 (m, 3H), 2.24 – 2.12 (m, 1H) ppm. ^13^C NMR (151 MHz, DMSO-*d*_*6*_): δ 177.20, 172.03, 75.85, 27.15, 25.81 ppm.

#### *tert*-Butyl (2*S*)-5-oxooxolane-2-carboxylate (2)

*N*,*N*′-Diisopropylcarbodiimide (8.60 mL, 54.9 mmol) was added to a stirring solution of lactone **1** (3.86 g, 27.7 mmol), *tert*-butanol (3.20 mL, 33.7 mmol) and 4-dimethylaminopyridine (0.339 g, 2.77 mmol) in dichloromethane (50 mL) and cooled in an ice bath. The reaction mixture was left to stir for 16 h, then solvent was removed *in vacuo*. The residue was taken up in 1:1 diethyl ether:hexane (100 mL), filtered, and evaporated *in vacuo*. The residue was purified by flash chromatography on silica (1:1 Et_2_O:Hex) to afford *tert*-butyl ester **2** as an off-white solid (4.72 g, 86%). [α]^25^_D_ =  + 12.7 (*c* 1.0, EtOH). ^1^H NMR (600 MHz, CDCl_3_): δ 4.84—4.79 (m, 1H), 2.66 – 2.57 (m, 1H), 2.57—2.46 (m, 2H), 2.32—2.21 (m, 1H), 1.50 (s, 9H) ppm. ^13^C NMR (151 MHz, CDCl_3_): δ 176.20, 169.00, 83.07, 76.27, 27.90, 26.76, 25.86 ppm. HRMS m/z: [M + NH_4_]^+^ calcd. for C_9_H_18_NO_4_^+^ 204.1236; found 204.1231.

#### Benzyl *tert*-butyl (2*S*)-2-hydroxypentanedioate (3)

1 M aqueous potassium hydroxide (10.0 mL, 10.0 mmol) was added to a stirring solution of *tert*-butyl ester** 2** (1.91 g, 10.2 mmol) in THF (10 mL). The reaction mixture was stirred at 40 °C for 2 h, then solvent was removed *in vacuo*. Dimethylformamide (20 mL) and benzyl bromide (1.30 mL, 10.9 mmol) were added to the residue, and the reaction mixture was left to stir for 16 h at ambient temperature. Water (50 mL) was added, and the reaction mixture was extracted with ethyl acetate (3 × 50 mL). The extracts were combined and evaporated *in vacuo*. The residue was purified by flash chromatography on silica (1:9 EtOAc:Hex) to afford alcohol **3** as a pale yellow oil (2.35 g, 78%). [α]^25^_D_ =  *− *6.60 (*c* 1.0, EtOH). ^1^H NMR (600 MHz, CDCl_3_): δ 7.39—7.28 (m, 4H), 5.13 (s, 2H), 4.13 – 4.03 (m, 1H), 2.88 (d, *J* = 5.4 Hz, 1H), 2.60—2.40 (m, 2H), 2.20—2.11 (m, 1H), 1.95—1.86 (m, 1H), 1.48 (s, 9H) ppm. ^13^C NMR (151 MHz, CDCl_3_): δ 173.91, 173.02, 135.96, 128.56, 128.22, 128.18, 82.79, 69.61, 66.34, 29.77, 29.50, 28.01 ppm. HRMS m/z: [M + H]^+^ calcd. for C_16_H_23_O_5_^+^ 295.1545; found 295.1526.

#### Benzyl *tert*-butyl (2*R*)-2-(1,4,7,10-tetraazacyclododecan-1-yl)pentanedioate (4)

Methanesulphonyl chloride (1.40 mL, 18.1 mmol) was added to a stirring solution of alcohol **3** (4.59 g, 15.6 mmol) and triethylamine (2.50 mL, 17.9 mmol) in anhydrous dichloromethane (60 mL) cooled in an ice bath. The reaction mixture was stirred for 30 min at ambient temperature, then washed with water (60 mL) and brine (60 mL). The organic phase was dried over magnesium sulphate, filtered, and evaporated to dryness *in vacuo*. The residue was dissolved in acetonitrile (60 mL) and added dropwise over 1 h to a stirring suspension of 1,4,7,10-tetraazacyclododecane (5.33 g, 31.0 mmol) and potassium carbonate (2.16 g, 15.6 mmol) in acetonitrile (120 mL). The reaction was stirred for 20 h at 50 °C, filtered, and the filtrate evaporated in vacuo. The residue was dissolved in ethyl acetate (250 mL), then washed with water (3 × 200 mL), brine (200 mL), and dried over magnesium sulphate. The extract was filtered, and solvent removed *in vacuo* to afford mono-alkylated **4** as a yellow oil (5.15 g, 74%). [α]^25^_D_ =  + 38.0 (*c* 1.0, EtOH). ^1^H NMR (600 MHz, CDCl_3_): δ 7.39—7.30 (m, 5H), 5.15—5.09 (m, 2H), 3.32 (dd, *J* = 8.5, 6.5 Hz, 1H), 2.96—2.43 (m, 18H), 2.18—2.06 (m, 1H), 1.97—1.82 (m, 1H), 1.46 (s, 9H) ppm. ^13^C NMR (151 MHz, CDCl_3_): δ 172.98, 171.57, 136.03, 128.53, 128.27, 128.21, 81.26, 66.24, 62.68, 48.90, 47.58, 46.05, 45.86, 30.93, 28.27, 24.33 ppm. HRMS m/z: [M + H]^+^ calcd. for C_24_H_41_N_4_O_4_^+^ 449.3128; found 449.3321.

#### Benzyl *tert*-butyl(2*R*)-2-(4,7,10-tris(2-amino-2-oxoethyl)-1,4,7,10-tetraazacyclododecan-1yl)pentanedioate (5)

Compound **4** (2.58 g, 5.76 mmol) was added to a mixture of 2-bromoacetamide (3.60 g, 26.1 mmol) and potassium carbonate (3.63 g, 26.1 mmol) in acetonitrile (60 mL), and left to stir for 20 h at 50 °C. The reaction mixture was filtered through celite, and solvent was removed *in vacuo*. The residue was dissolved in water (20 mL) and extracted with dichloromethane (5 × 50 mL). The combined organic extracts were dried over magnesium sulphate, filtered, and solvent removed *in vacuo*. The residue was triturated with diethyl ether (20 mL), filtered, and purified by preparative HPLC using a gradient of water and acetonitrile to afford compound **5** as a white solid (2.37 g, 66%). [α]^25^_D_ =  *− *55.0 (*c* 1.0, EtOH)*.* ^1^H NMR (500 MHz, CDCl_3_): δ 7.59 (bs, 2H), 7.42—7.29 (m, 5H), 6.94 (bs, 2H), 6.47 (bs, 2H), 5.13 (s, 2H), 3.32 – 3.27 (m, 1H), 3.20 – 2.29 (m, 24H), 2.03—1.94 (m, 1H), 1.89—1.81 (m, 1H), 1.44 (s, 9H) ppm. ^13^C NMR (151 MHz, CDCl_3_): δ 174.31, 173.81, 172.87, 171.44, 135.79, 128.64, 128.40, 128.34, 81.69, 66.50, 61.83, 58.98, 58.23, 54.65, 53.70, 53.66, 50.58, 30.95, 28.32, 24.74 ppm. HRMS m/z [M + H]^+^ calcd. for C_30_H_50_N_7_O_7_^+^ 620.3772; found 620.3855.

#### (4***R***)-5-***tert***-Butoxy-5-oxo-4-(4,7,10-tris(2-amino-2-oxoethyl)-1,4,7,10-tetraazacyclododecan-1-yl)pentanoic acid (DOTAMGA-O.^***t***^Bu)

10% w/w palladium on carbon (0.143 g) was added to a solution of compound **5** (1.43 g, 2.30 mmol) and ammonium formate (0.578 g, 9.17 mmol) in methanol (40 mL). The atmosphere was purged with nitrogen, and the reaction stirred at reflux for 4 h. The reaction mixture was filtered through celite, and solvent removed *in vacuo* to afford DOTAMGA-O^*t*^Bu as a pale yellow solid (1.22 g, 100%)**.** [α]^25^_D_ =  *− *23.3 (*c* 1.0, EtOH). ^1^H NMR (600 MHz, DMSO-*d*_*6*_): δ 7.64 (s, 2H), 7.58 (s, 1H), 7.13 (s, 2H), 7.08 (s, 1H), 3.39 – 2.54 (m, 23H), 2.35—2.22 (m, 2H), 1.91—1.81 (m, 1H), 1.78—1.65 (m, 1H), 1.42 (s, 9H) ppm. ^13^C NMR (151 MHz, DMSO-*d*_*6*_): 174.58, 173.21, 171.95, 171.72, 81.11, 63.10, 56.69, 54.08, 52.93, 51.98, 49.04, 48.93, 31.15, 28.30, 24.79. HRMS m/z: [M + H]^+^ calcd. for C_23_H_44_N_7_O_7_^+^ 530.3302; found 530.3374.

### Peptide synthesis

TCMC-MC1RL and DOTAMGA-MC1RL were synthesised using Fmoc-based solid phase peptide synthesis protocols. Fmoc protected Rink amide resin (0.20 g, 0.10 mmol, 0.49 mmol/g, 1 eq.) was treated with 20% piperidine in DMF (5 mL) for 15 min with intermittent stirring, then washed with DMF (5 × 5 mL), DCM (5 × 5 mL), and DMF (5 × 5 mL). A solution of Fmoc-Lys(Dde)-OH (0.27 g, 0.50 mmol, 5 eq.), HCTU (0.21 g, 0.50 mmol, 5 eq.) and DIPEA (0.17 mL, 1.0 mmol, 10 eq.) in DMF (5 mL) was added to the resin, and stirred intermittently for at least 1 h at ambient temperature. The resin was filtered, and washed with DMF (5 × 5 mL), DCM (5 × 5 mL), and DMF (5 × 5 mL). The deprotection and coupling steps were repeated with appropriate *N*-Fmoc protected amino acids until deprotection of the histidine residue, then 4-phenylbutyric acid (0.082 g, 0.50 mmol, 5 eq.) was coupled analogously to the amino acids. The lysine Dde group was cleaved upon treatment with 1% hydrazine in DMF (2 × 5 mL, 5 min each), and the resin was washed and filtered as previously described. Fmoc-aminohexanoic acid (0.18 g, 0.50 mmol, 5 eq.) was coupled then deprotected, before coupling of the appropriate bifunctional chelator. For TCMC-MC1RL and DOTAMGA-MC1RL, this was DOTAM-mono-acid (0.20 g, 0.50 mmol, 5 eq.), and DOTAMGA-O^*t*^Bu (0.26 g, 0.50 mmol, 5 eq.) respectively. Resin cleavage and global deprotection was achieved upon treatment with 92.5:2.5:2.5:2.5 TFA:H_2_O:DODT:TIPS (10 mL) with agitation for 4 h at ambient temperature. The resin was filtered, and the filtrate concentrated to approximately 0.5 mL under a stream of nitrogen gas. The peptides were precipitated in diethyl ether (20 mL), cooled in an acetone/dry ice bath for 10 min, then centrifuged (3450 × *g*, 10 min). The supernatant was discarded, and the precipitated peptides dissolved in 30% acetonitrile in water and lyophilised. The crude peptides were purified by preparative HPLC with a gradient of water and acetonitrile with 0.1% trifluoracetic acid, then lyophilised to afford the products as white solids. Characterisation data for TCMC-MC1RL and DOTAMGA-MC1RL are reported in Table S1 and Figures S8 and S9 of the supplementary information.

### Receptor affinity assays

SK-MEL-13 cells overexpressing either MC1R or melanocortin 4 receptor (MC4R) were seeded in a 96 well plate in 100 µL culture medium (RPMI 1640 (Thermo Fisher), complemented with 10% fetal bovine serum (Thermo Fisher) and 1% penicilin/streptomycin (Thermo Fisher)), and incubated for at least 16 h at 37 °C. The culture medium was removed, and 80 µL assay buffer (RPMI 1640 medium without phenol red (Thermo Fisher) with 20 mM HEPES (Thermo Fisher), 0.25% BSA (Sigma Aldrich), and 1 mM 1,10 phenanthroline (Sigma Aldrich)) containing [^125^I]I-NDP-MSH (0.1 nM, 1 kBq/mL, Revvity) and varying concentrations of ligand were added to each well in triplicate. Cells incubated without ligand, and without both ligand and [^125^I]I-NDP-MSH served as positive and negative controls respectively. The plates were then incubated for 2 h at ambient temperature, and the assay buffer was removed. Cells were washed with assay buffer (200 µL) and PBS containing 0.25% BSA (200 µL, Thermo Fisher), then Ultima Gold XR liquid scintillation cocktail (200 µL, Revvity) was added. Scintillation was measured over 1 min with a scintillation counter (MicroBeta TriLux 1450, Wallac) and expressed in CPM. The average of the negative controls was subtracted from all counts, and each count expressed as a percentage relative to the averaged positive controls. These were fitted to a three parameter dose response curve in GraphPad Prism version 9 to obtain IC_50_ values.

### UV–Vis kinetic studies

Kinetic studies were performed with UV–Vis spectrophotometry using a Cary 5000 UV–Vis-NIR spectrophotometer (Agilent). A solution containing 0.02 mM ligand and 0.01 mM PbCl_2_ in 0.1 M sodium acetate balanced to pH = 5.5 with 1 M HCl was prepared in a quartz cuvette. The spectrometer was baselined after the addition of peptide and sodium acetate. UV–Vis absorbance spectra were collected at intervals immediately after the addition of the PbCl_2_ in the range of 200–350 nm.

### Radiolabelling

For the radiolabelling study, 0.5 MBq ^212^PbCl_2_ was added to varying concentrations of ligand in 0.1 M sodium acetate at pH = 5.5 (100 µL), and heated at 30 °C with agitation for 20 min. Radiochemical conversions were determined by analysing an aliquot of the reaction by iTLC-SA (Agilent), which was developed with 25 mM ethylenediaminetetraacetic acid (EDTA) at pH = 5. Under these conditions, radiolabelled peptides remained at the baseline, while unbound radionuclides eluted with the solvent front. The TLC plate was halved crosswise, and the activity of ^203^Pb or ^212^Pb in each half quantified by gamma spectroscopy from their respective 279 and 239 keV γ-emissions. The radiochemical conversion was determined by dividing the radionuclide activity of the top half by that of the bottom half. Reactions were performed in triplicate, and RCCs expressed as mean ± standard deviation.

Radiolabelling for subsequent in vitro and in vivo studies were performed analogously in 0.1 M sodium acetate at pH = 5.5 with various amounts of ligand and either ^203^Pb or ^212^Pb. For the biodistribution experiments, the radiolabelling solution was then formulated with the addition of sodium ascorbate and DTPA, such that the final concentrations of sodium acetate, sodium ascorbate, and DTPA were 0.05 M, 0.25 M, and 0.25 mM respectively. Radio-HPLC chromatograms for all radiolabelled compounds are reported in Figures S10 to S13 of the supplementary information.

### Lipophilicity

Radiolabelling was performed as described above using 0.5 MBq ^212^Pb and 0.40 nmol peptide in 0.1 M sodium acetate buffer (200 µL). An aliquot of the reaction mixture (10 µL) was added to 0.1 M phosphate buffer pH = 7.4 (90 µL), then 1-octanol (100 µL) was added. The mixture was vortexed for 1 min, and the phases separated by centrifugation. To ensure unbound ^212^Pb did not interfere with the determination of the partition coefficient, an aliquot (10 µL) of each phase was spotted onto an iTLC-SA plate (Agilent) and developed with 25 mM EDTA at pH = 5. The plates were halved crosswise, and the activity of ^212^Pb in the bottom halves (corresponding to the bound radionuclides) quantified by gamma spectroscopy. The distribution coefficient (D_7.4_) was calculated as the activity of ^212^Pb in the octanol plate divided by that of the aqueous plate. Experiments were performed in triplicate, and the mean distribution coefficients expressed logarithmically as log D_7.4_.

### Serum stability studies

For the radio-TLC serum stability studies, 10 µg of each peptide was radiolabelled as described above with 2 MBq of ^212^Pb in 200 µL sodium acetate buffer. ^212^Pb. The reactions were diluted fourfold in human serum (Sigma), and incubated at 37 °C. At 0, 5, 24, and 48 h after radiolabelling, an aliquot of each reaction was taken and spotted onto an iTLC-SA plate (Agilent) and developed with 25 mM EDTA at pH = 5. The plate was cut in half crosswise, and the activity of ^212^Pb present in each half quantified by gamma spectroscopy from its 239 keV γ-emission. The fraction of ^212^Pb remaining bound at each time point was then determined by dividing the measured activity of the top half by that of the bottom half.

For the radio-HPLC serum stability studies, compounds were radiolabelled using 2.5 MBq of ^203^Pb and 6.3 nmol peptide, in 0.1 M sodium acetate (25 µL) as described above. The radiolabelled peptides (10 µL, 1 MBq) were added to either human serum (Sigma) (90 µL) or 0.1 M phosphate buffered saline pH = 7.4 (90 µL), and incubated at 37 ºC with agitation. After 0, 2, and 20 h, an aliquot of the reaction (30 µL) was added to a solution of acetonitrile (45 µL) and incubated at 0 °C for 15 min. Precipitate was pelletised by centrifugation, and the supernatant was taken and analysed by radio-HPLC. Radiochemical purity was determined at each time point by integrating the radio-HPLC chromatogram.

### In Vivo* Biodistribution*

*All animal studies were reviewed and approved by the University of Queensland Molecular Biosciences Animal Ethics Committee*. C57BL/6 mice were subcutaneously injected with B16F0 murine melanoma (7.5 × 10^5^ cells in 100 µL PBS) in the right flank which were allowed to grow for 11 days. Peptides (12.5 µg) were radiolabelled with ^203^Pb (2.5 MBq) in sodium acetate buffer (250 µL) and formulated as described above. Mice were injected with the formulated ^203^Pb labelled peptides (ca. 0.1 MBq in 100 µL 0.05 M sodium acetate, 0.25 M sodium ascorbate, and 0.25 mM DTPA, intravenous injection). Activities of the syringes were measured before and after injection to accurately calculate the dose administered. At predetermined time points, mice were euthanised and dissected. Organs were weighed, and the activity of ^203^Pb in each quantified using a gamma counter (Gamma Wizard 2480, PerkinElmer) from its 279 keV γ-emission. Measured activities were decay corrected to injection time, and doses in each organ were determined using a standard curve. The dose in each organ was calculated relative to the injected dose (%ID/organ), which was divided by the organ weight to give the percentage injected dose per gram (%ID/g). These values are reported in Tables S3 and S4 of the supplementary information.

### Statistical analysis

Pairwise comparisons of experimental data were analysed using an unpaired *t*-test with GraphPad Prism version 9. *P* values below 0.05 were considered statistically significant.

## Supplementary Information

Additional file1 (PDF 829 KB)

## Data Availability

All data generated or analysed during this study are included in this published article and its supplementary information files.

## Abbreviations

AUC: Area under curve
DOTA: 1,4,7,10-Tetraazacyclododecane-1,4,7,10-tetraacetic acid
DOTAGA: 2-(4,7,10-Tris(carboxymethyl)-1,4,7,10-tetraazacyclododecan-1-yl)pentanedioic acid
DOTAMGA: 2-(4,7,10-Tris(2-amino-2-oxoethyl)-1,4,7,10-tetraazacyclododecan-1-yl)pentanedioic acid
DO3AM: 4,7,10-Tris(2-amino-2-oxoethyl)-1,4,7,10-tetraazacyclododecan-1-yl)acetic acid
DTPA: Diethylenetriaminepentaacetic acid
EDTA: Ethylenediaminetetraacetic acid
MC1R: Melanocortin 1 receptor
MC4R: Melanocortin 4 receptor
PBS: Phosphate buffered saline
PSC: Pb specific chelator
RCC: Radiochemical conversion
r.t: Ambient temperature
SPECT: Single photon emission tomography
TCMC: 1,4,7,10-Tetraazacyclododecane-1,4,7,10-tetraacetic amide
TRT: Targeted radionuclide therapy
