# Supplementary material for: Synthesis and in vivo evaluation of a bifunctional, glutamic acid derived chelator for the 203/212Pb theranostic pair
Source: EJNMMI Radiopharm Chem. 2026 Jul 4;11:72. doi: 10.1186/s41181-026-00473-9 (PMC13616893; doi:10.1186/s41181-026-00473-9)
Supplement: Supplementary file 1 [file 41181_2026_473_MOESM1_ESM.pdf]

Supplementary Information

Synthesis and *in vivo* evaluation of a bifunctional, glutamic acid derived chelator for the $^{203/212}$Pb theranostic pair.

Jarred Michael Scaffidi-Muta[a,b], Kwong Ching Li[b], Didier Boucher[b], Thomas Kryza[b], William Tieu[b], Andrew David Abell[a]

[a] School of Physics, Chemistry & Earth Sciences, Adelaide University, Adelaide, South Australia, Australia

[b]AdvanCell Pty Ltd., Richlands, Queensland, Australia

Table of Contents

S1. Radiostability **2**

S2. NMR Spectra **3**

S3. HPLC Chromatograms **9**

S4. Peptide Characterisation and Purity **11**

S5. *Ex Vivo* Biodistribution **12**

S1. Radiostability

Compounds were radiolabelled with ^{212}Pb at 2.0 MBq/µg in 0.05 M sodium acetate, and either left unformulated, or were formulated with 0.25 M sodium ascorbate. The ^{212}Pb-labelled peptides were then left at ambient temperature and analysed by radio-HPLC at 0, 4 and 24 hours after radiolabelling. Radiochemical purity was determined at each time point by dividing the integration of the peak corresponding to the ^{212}Pb-labelled peptide by the total area under the chromatogram. Radiostability was then calculated by dividing the radiochemical purities by that at t = 0. Unformulated [^{212}Pb]Pb-TCMC-MC1RL was readily degraded, with only 14% of the intact compound remaining after an 18 hour incubation (Figure S1). In contrast, the addition of sodium ascorbate as a radioprotectant greatly improved stability, with formulated [^{212}Pb]Pb-DOTAMGA-MC1RL remaining virtually unchanged over 24 hours. Radiolabelled compounds were thus formulated with sodium ascorbate during the subsequent biodistribution experiments.

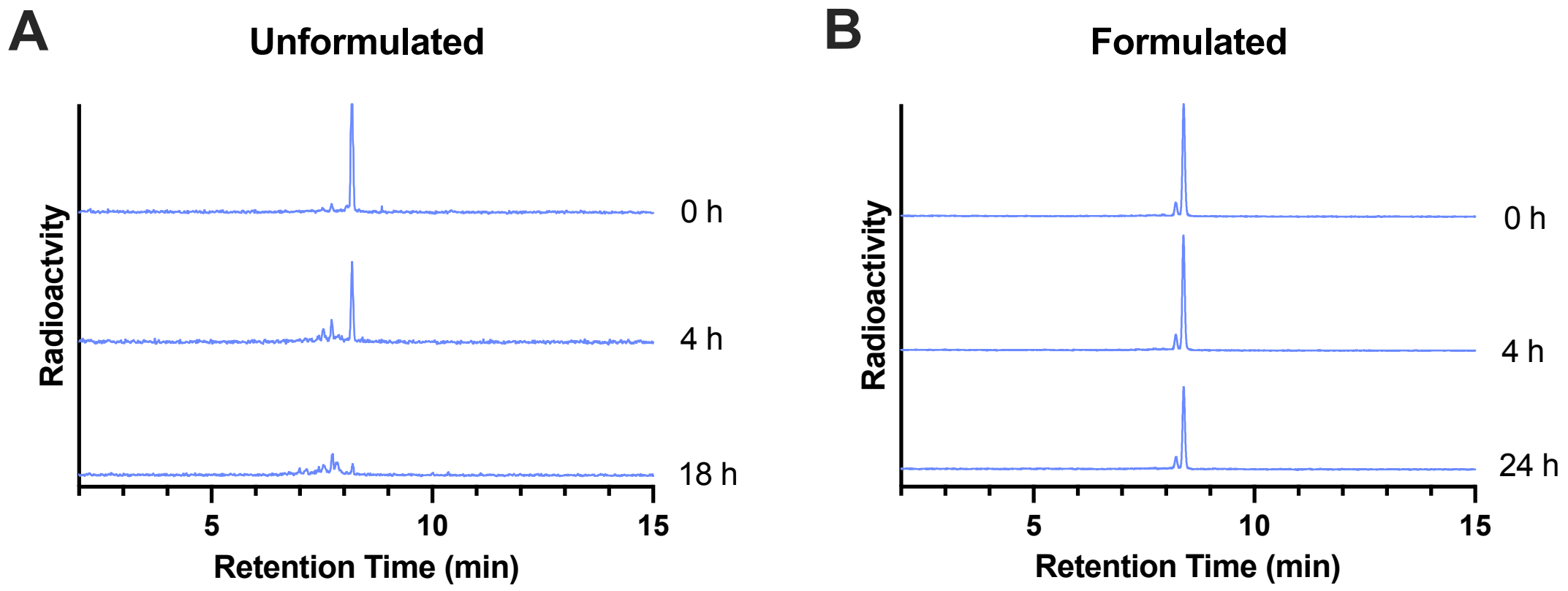

Figure S1: Radiostabilities of MC1R targeted peptides. (A) [^{212}Pb]Pb-TCMC-MC1RL incubated in 0.05 M NaOAc. (B) [^{212}Pb]Pb-DOTAMGA-MC1RL incubated in 0.05 M NaOAc with 0.25 M sodium ascorbate. HPLC chromatograms were acquired using a Shimadzu 40 Series HPLC system equipped with a radio-detector and a 150 × 4.6 mm Phenomenex Jupiter Proteo C18 HPLC column. Samples were eluted with a gradient of 10% to 50% acetonitrile in water with 0.1% TFA from 2 to 15 minutes at 2.0 mL/min.

S2. NMR Spectra

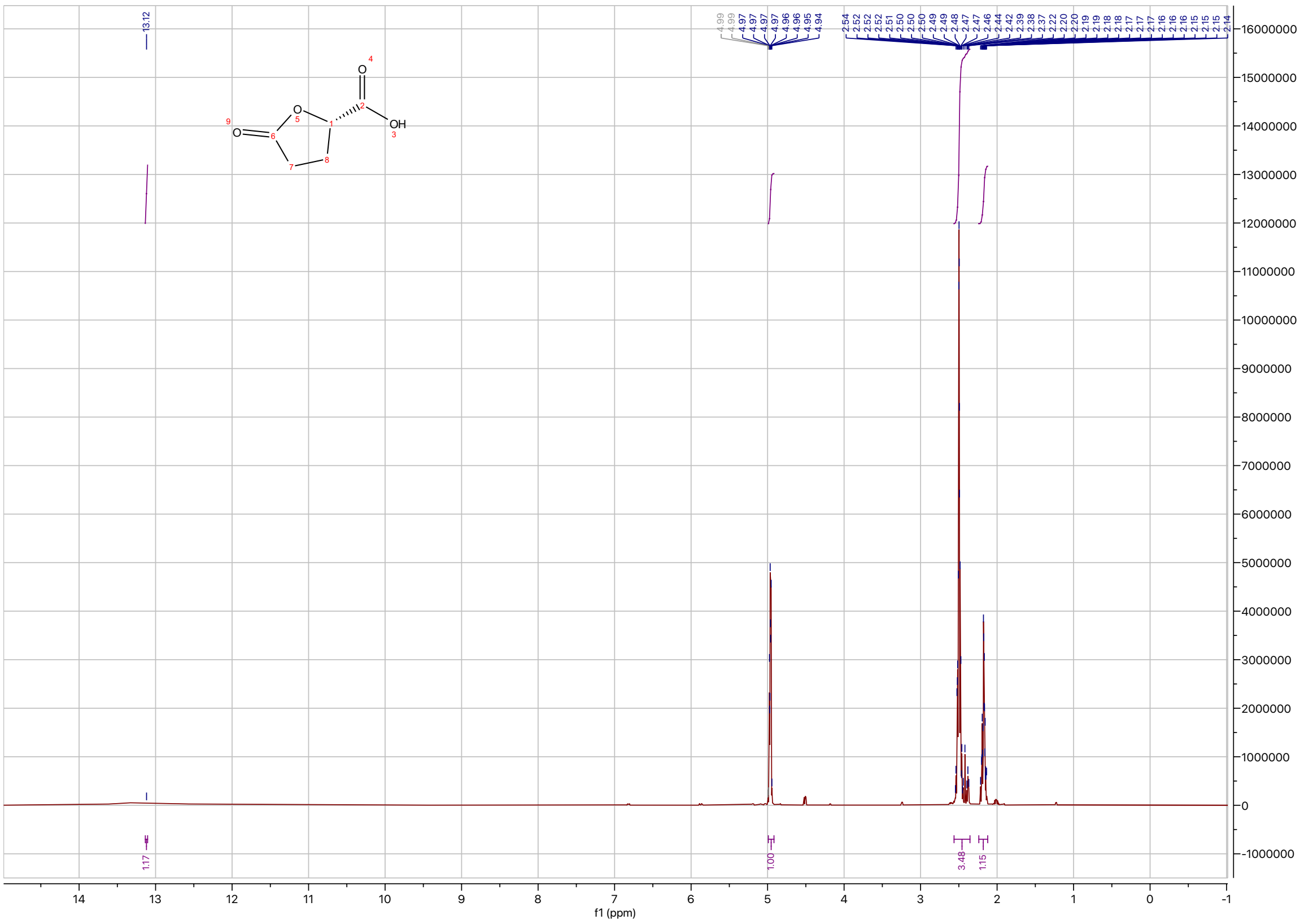

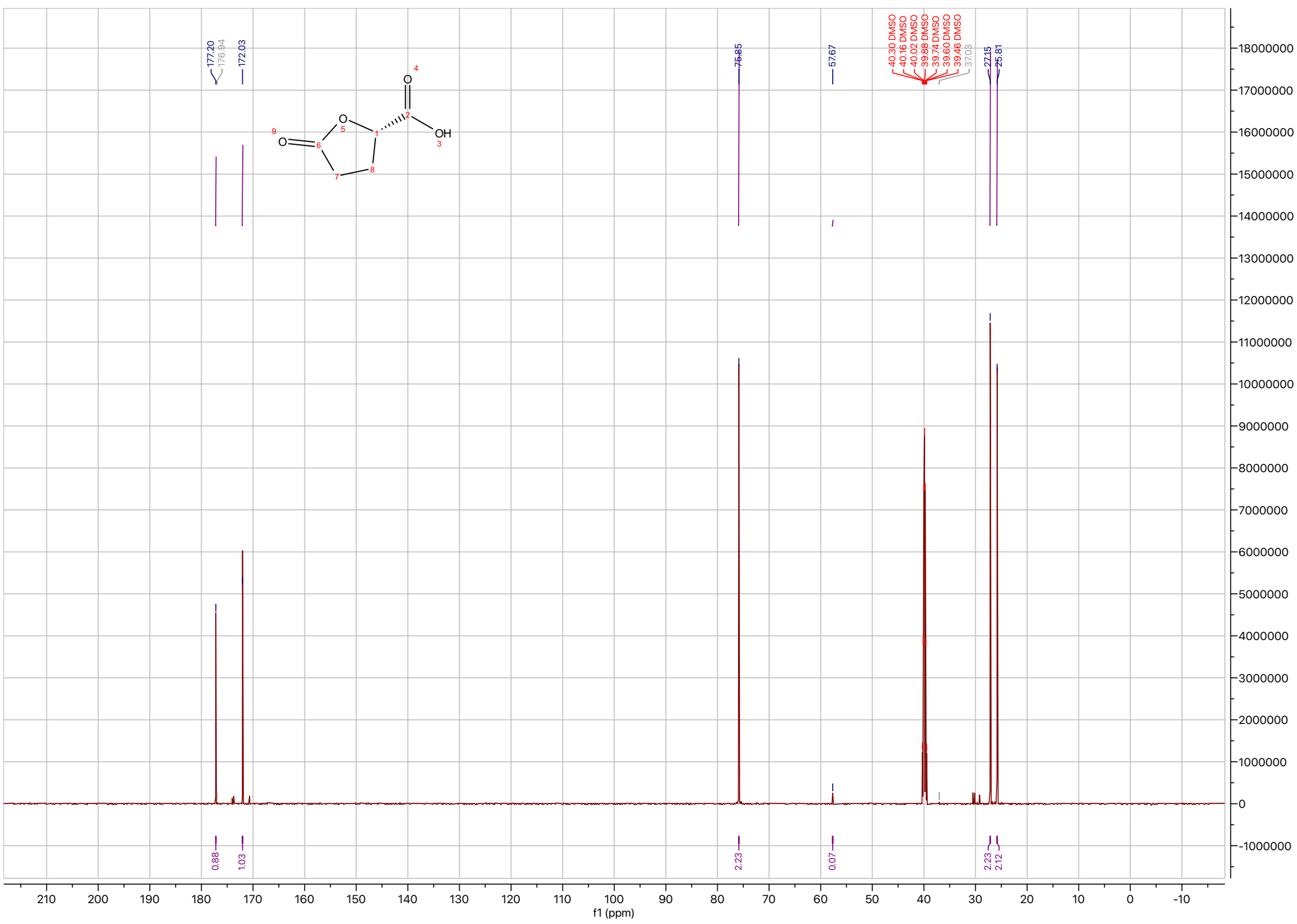

Figure S2: ^{1}H & ^{13}C NMR spectra of compound **1** in DMSO at ambient temperature.

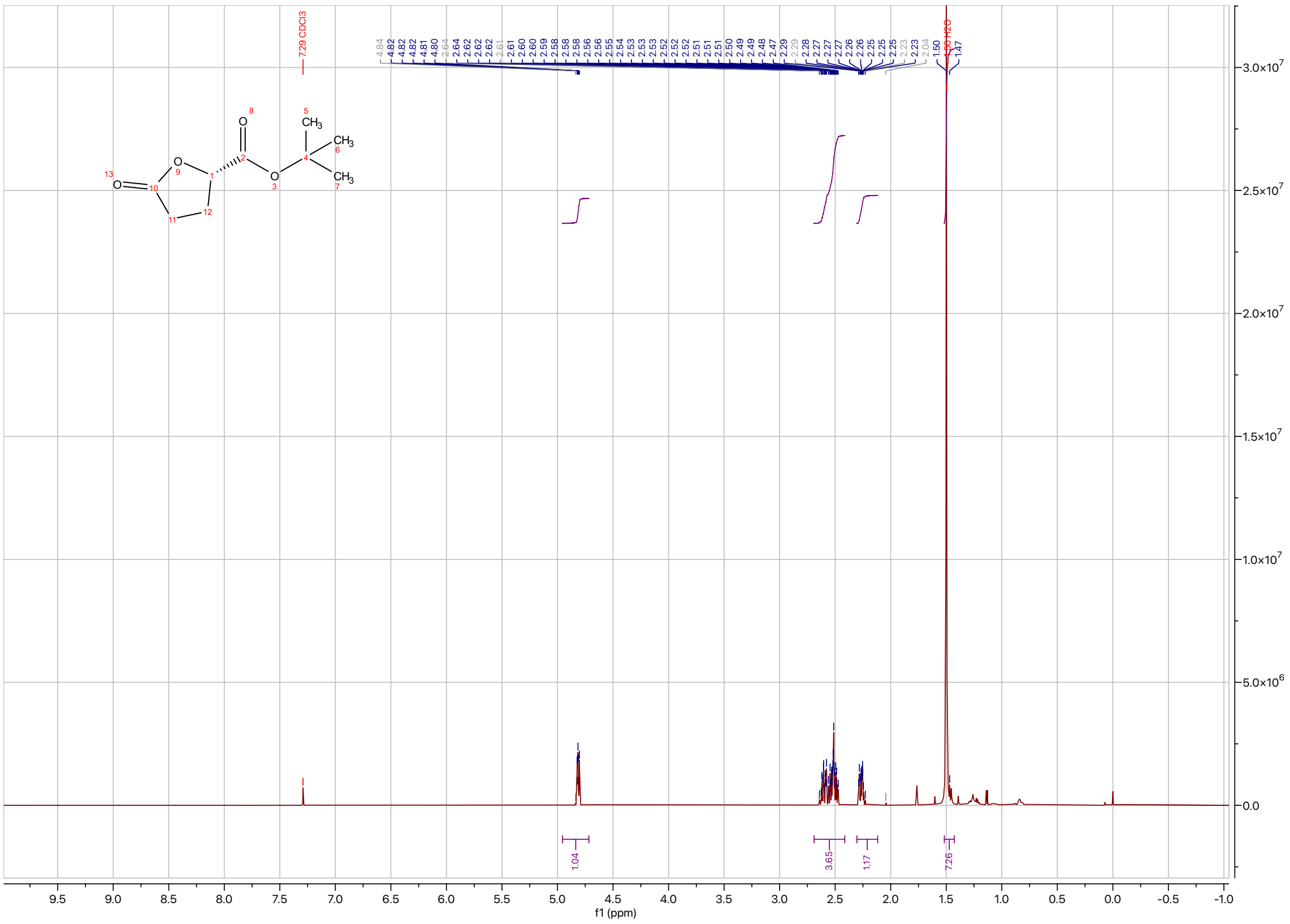

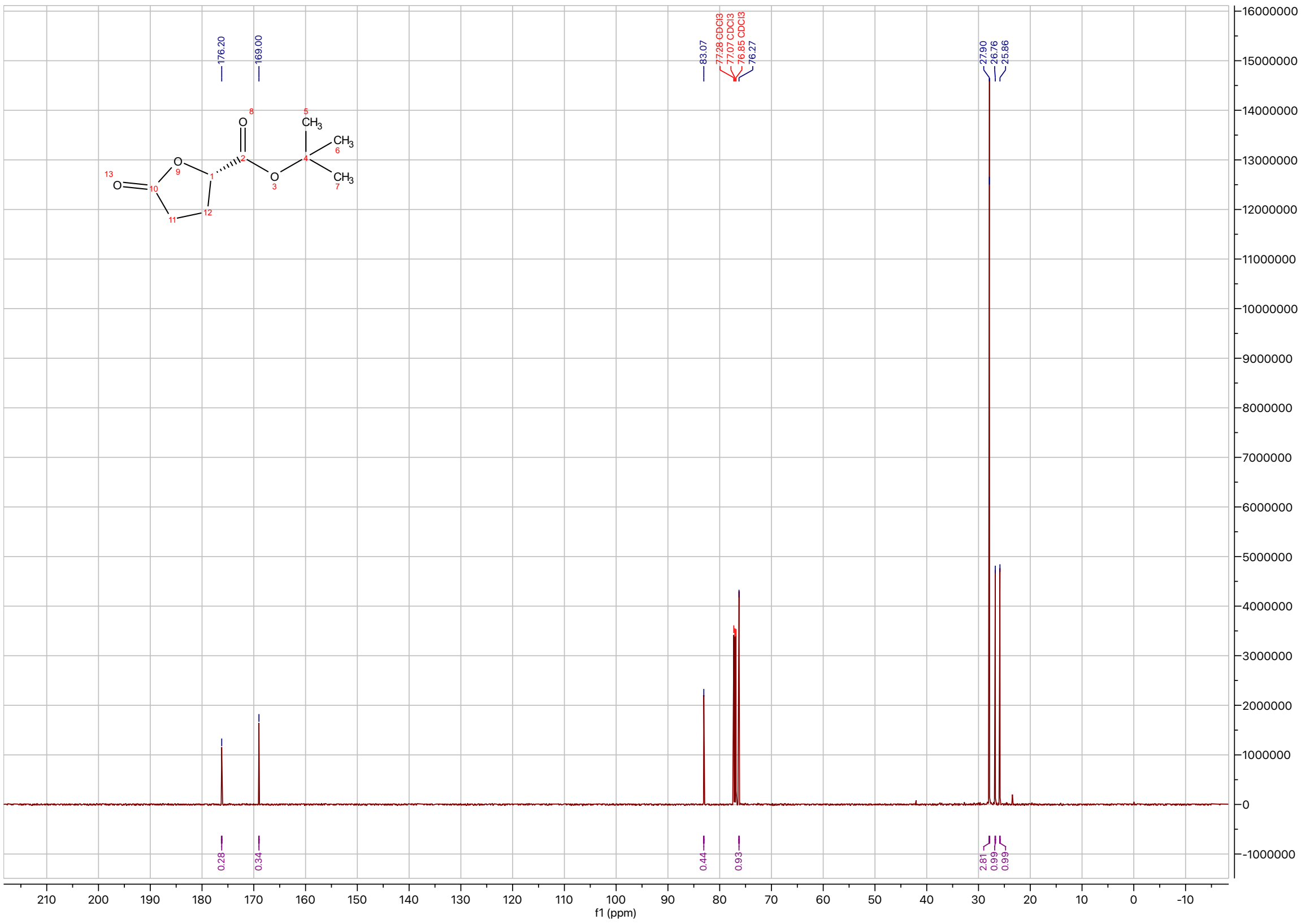

Figure S3: ^{1}H & ^{13}C NMR spectra of compound **2** in $CDCl_3$ at ambient temperature.

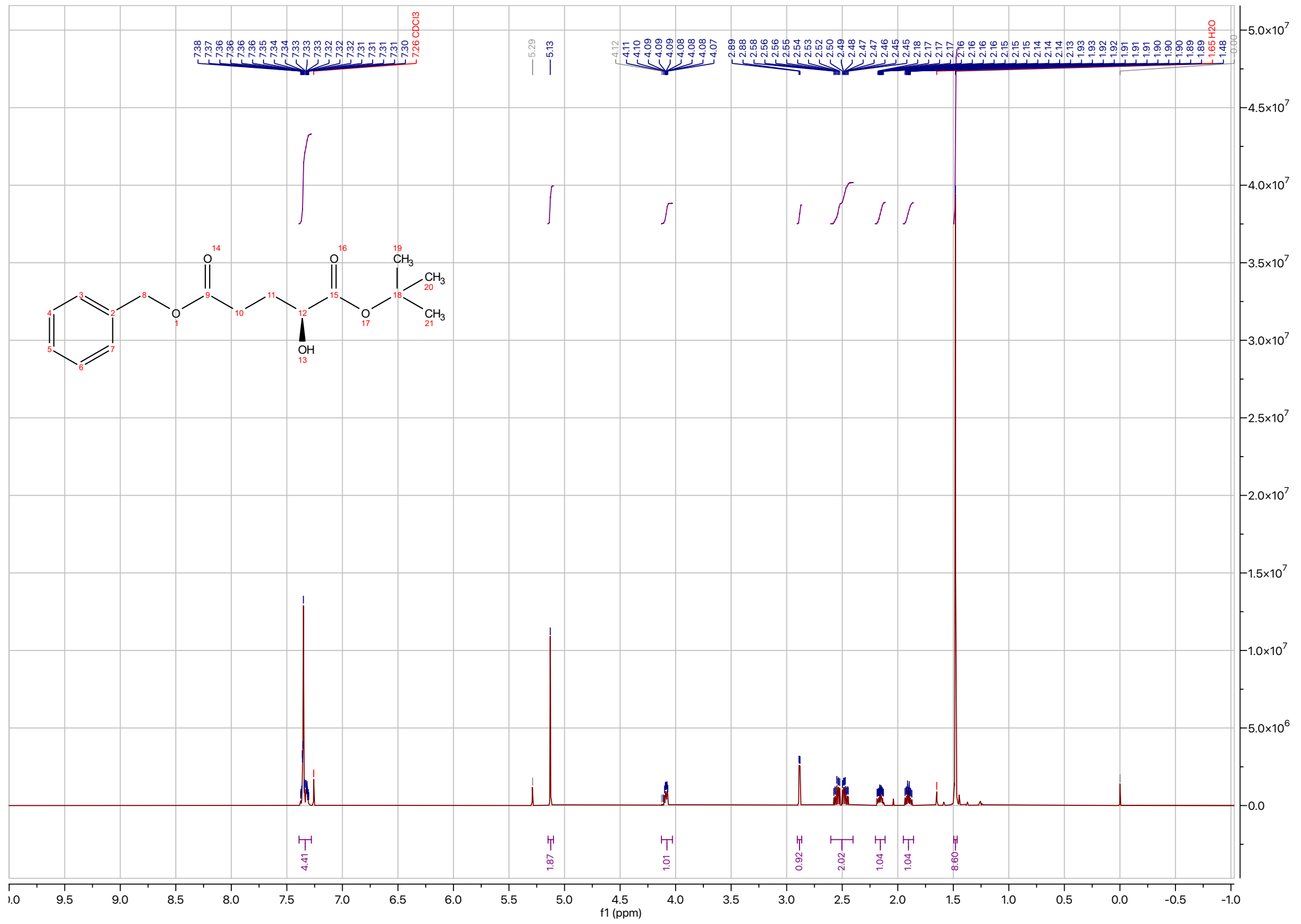

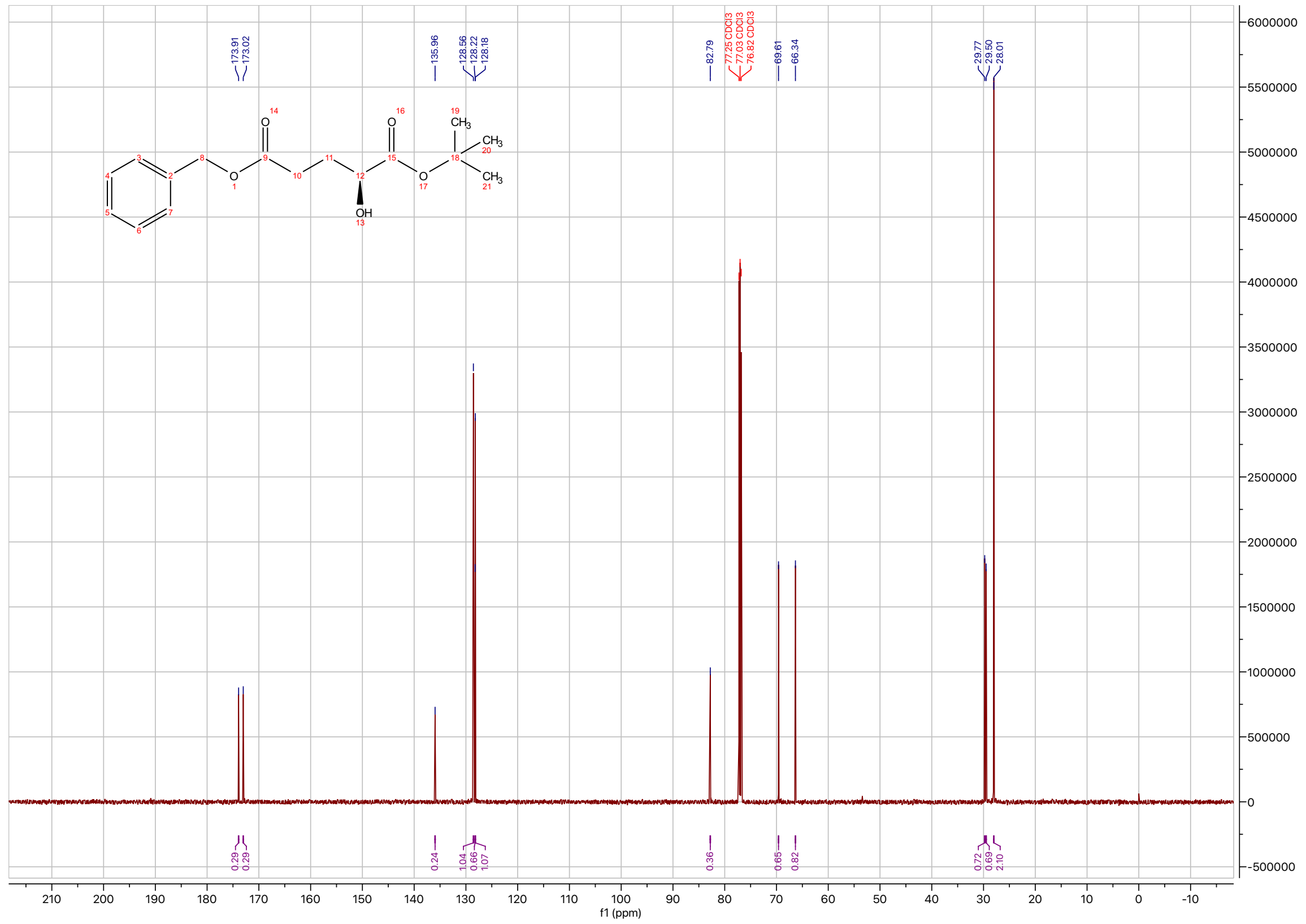

Figure S4: ^{1}H & ^{13}C NMR spectra of compound **3** in $CDCl_3$ at ambient temperature.

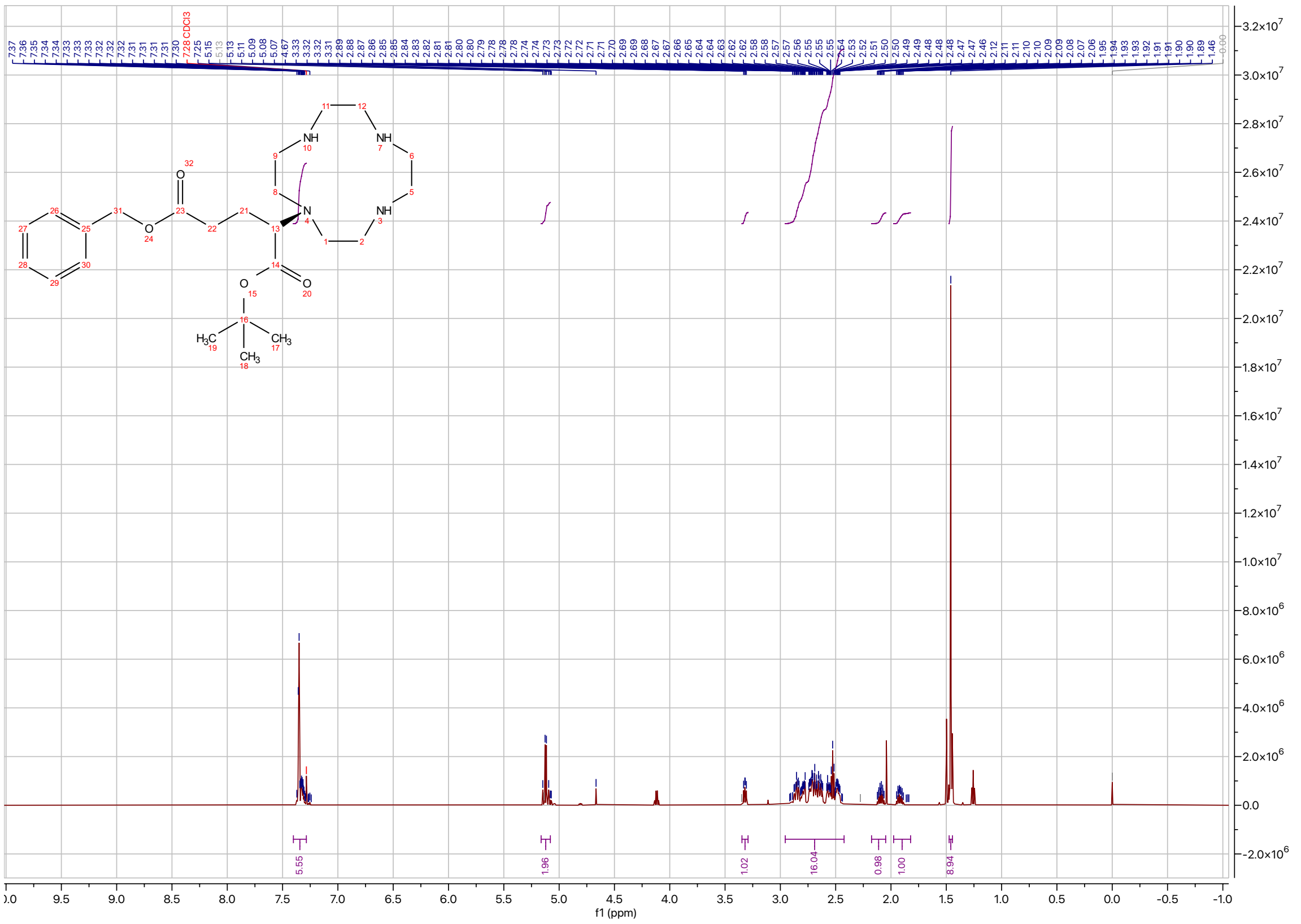

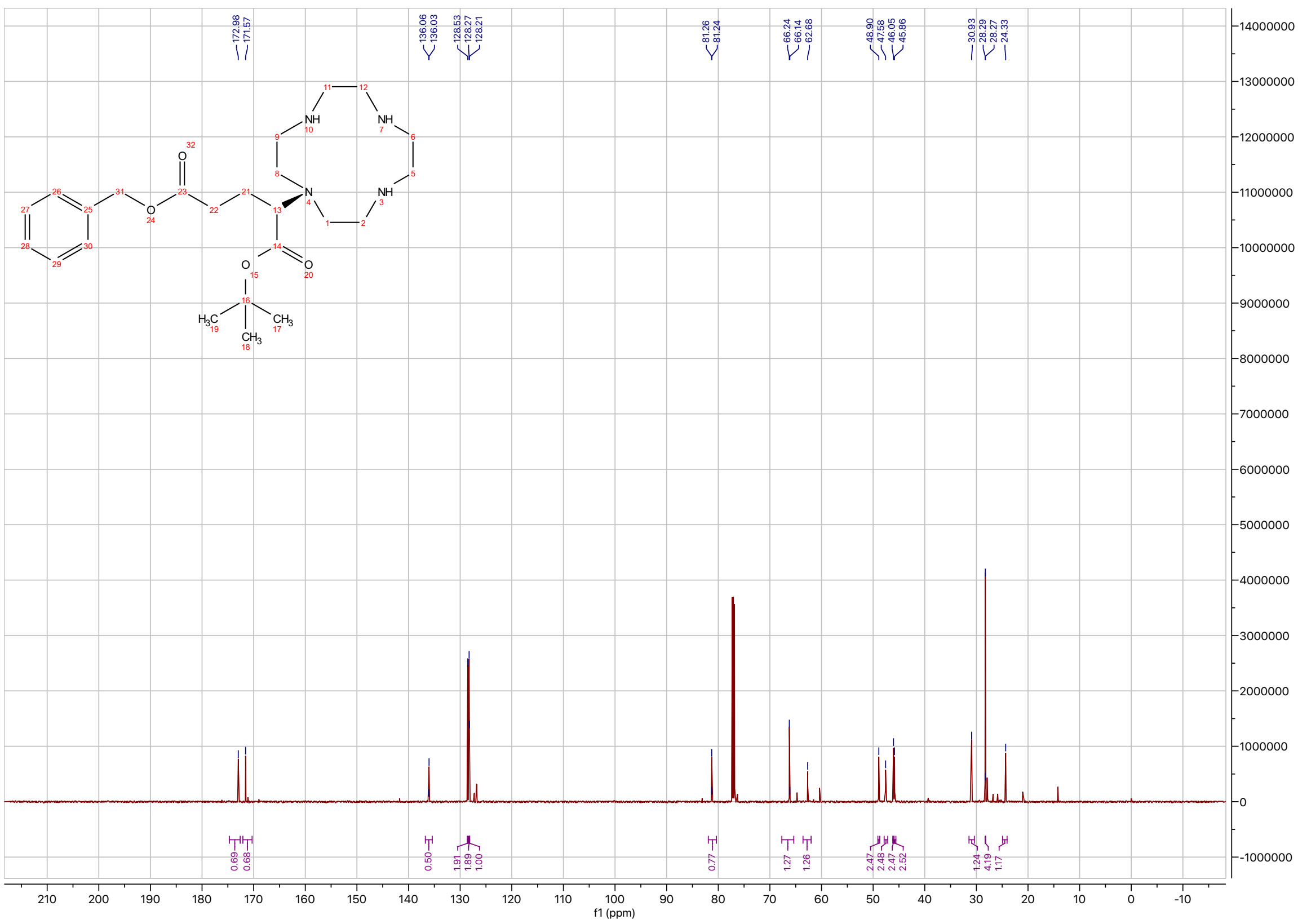

Figure S5: ^{1}H & ^{13}C NMR spectra of compound **4** in $CDCl_3$ at ambient temperature.

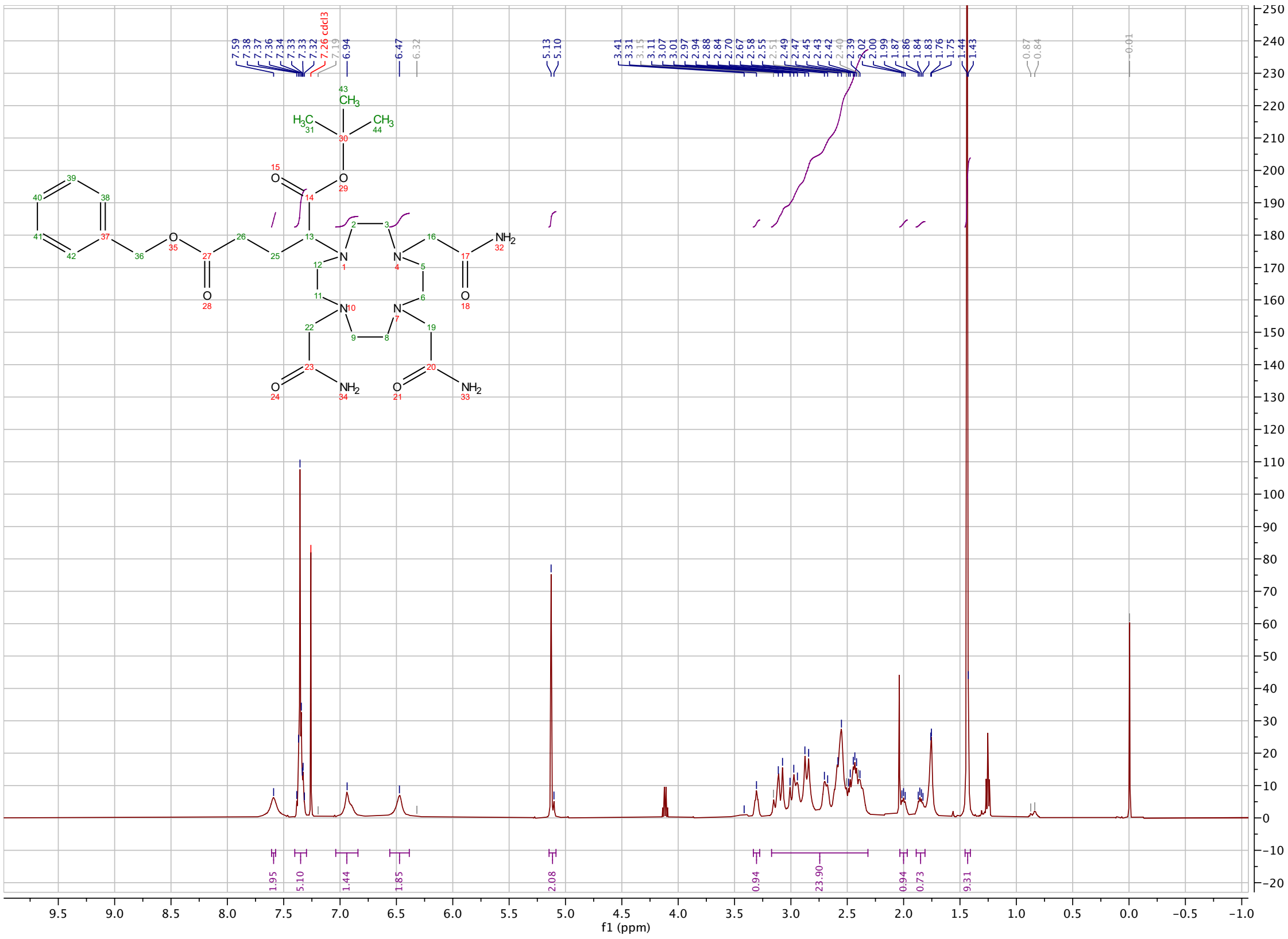

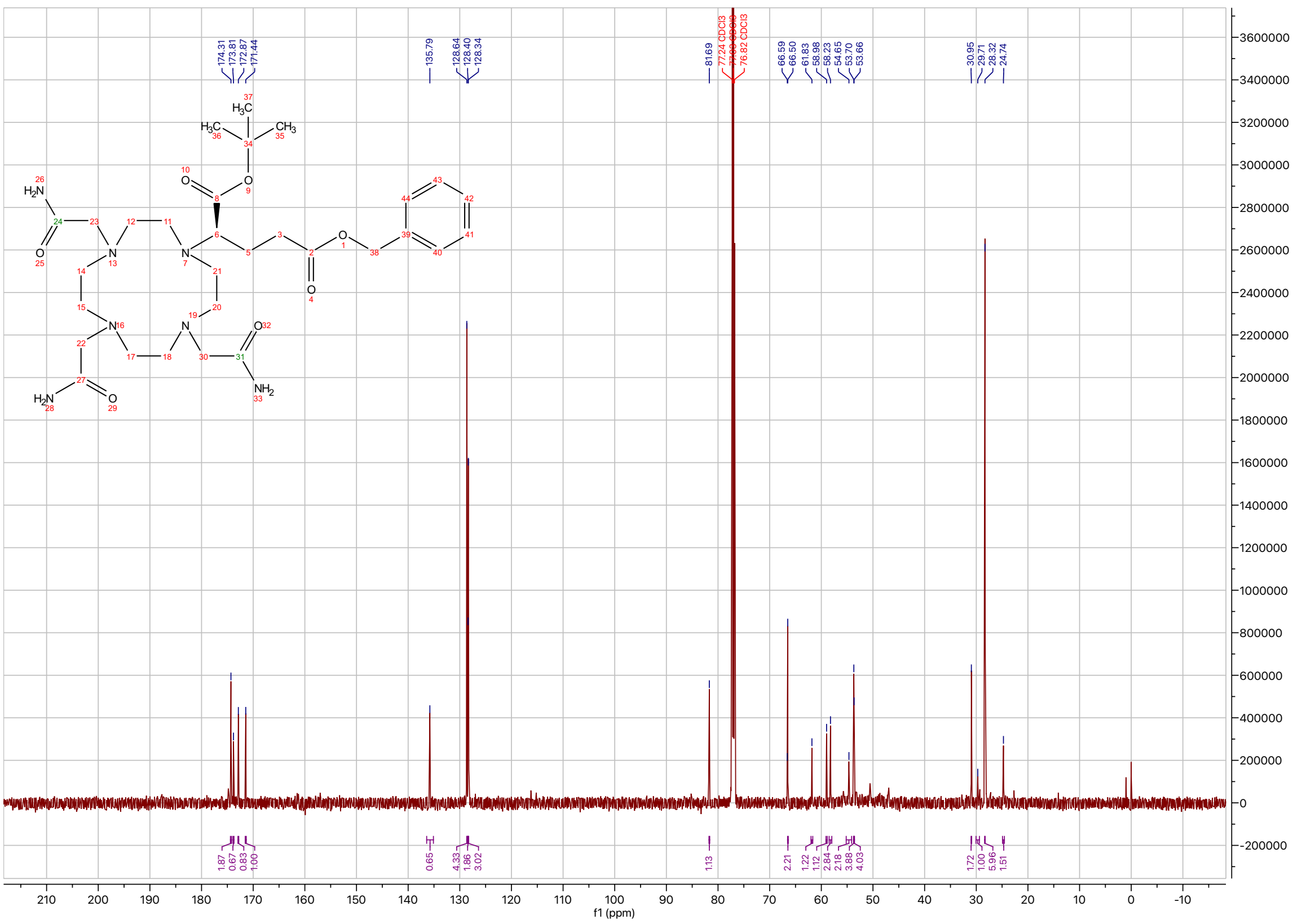

Figure S6: ^1H & ^{13}C NMR spectra of compound **5** in $CDCl_3$ at ambient temperature.

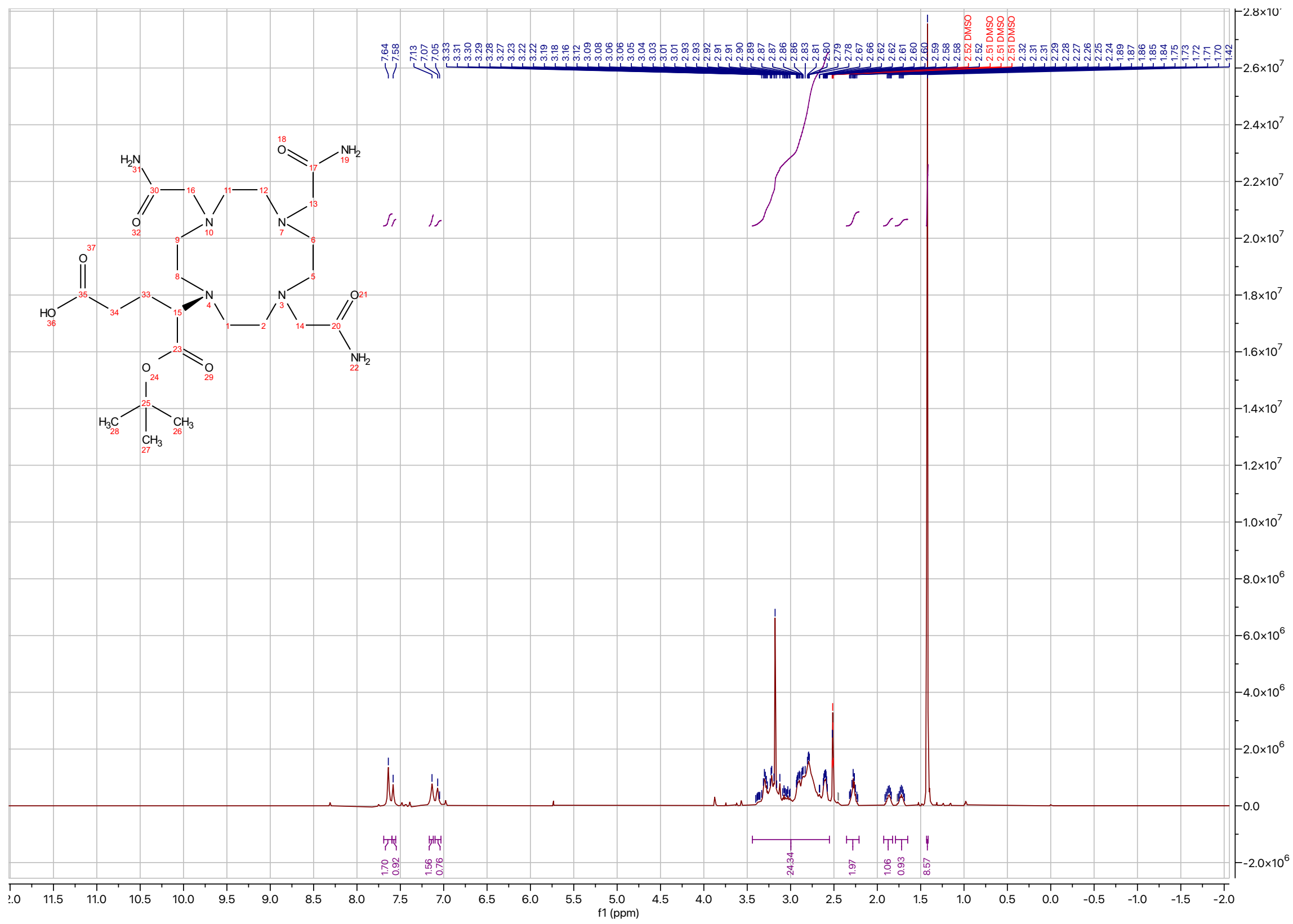

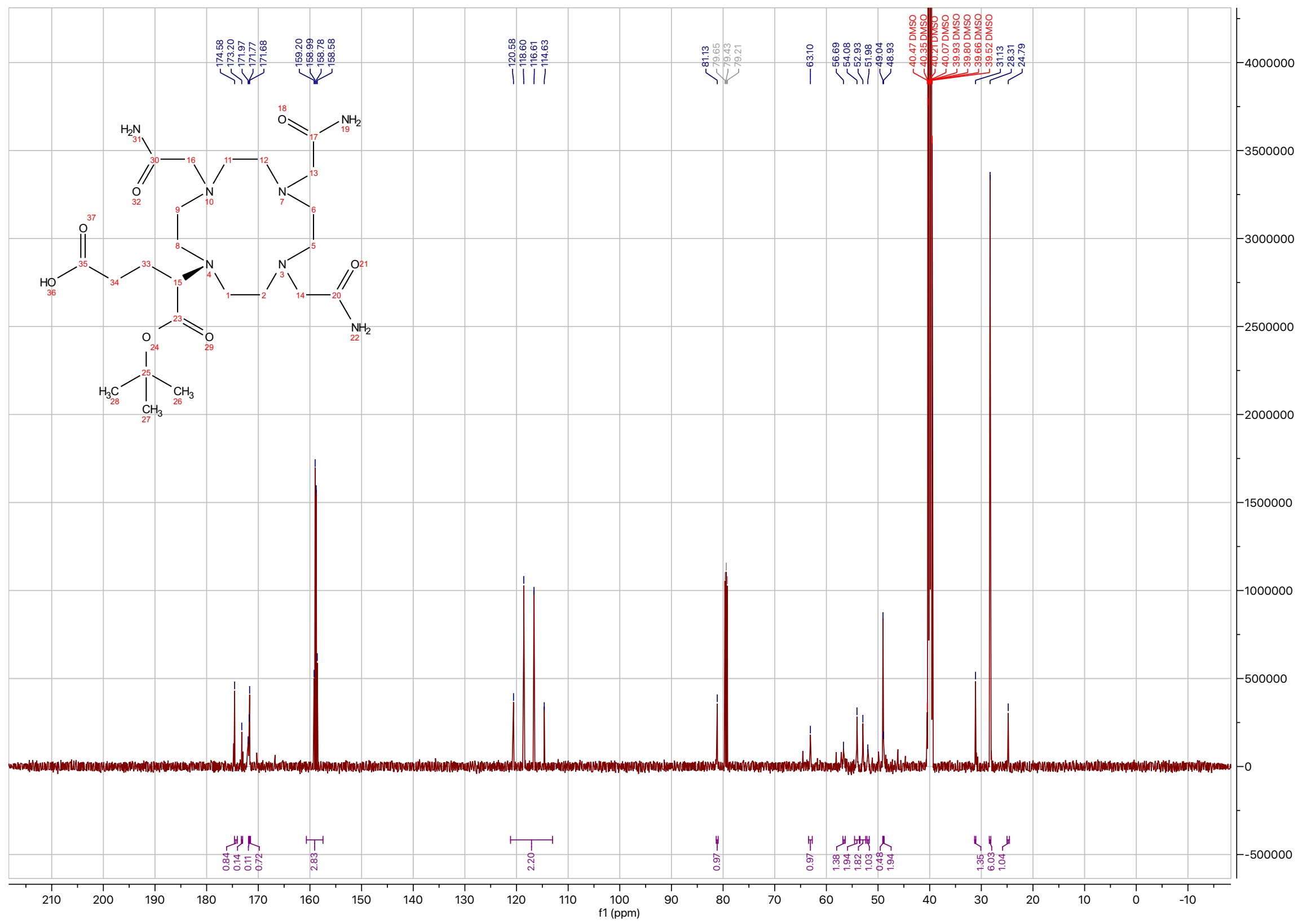

Figure S7: ^{1}H & ^{13}C NMR spectra of DOTAMGA-O^{t}Bu (TFA Salt) in DMSO at ambient temperature.

S3. HPLC Chromatograms

HPLC Method A: HPLC chromatograms were acquired using an Agilent 1260 infinity HPLC system equipped with a 250 × 4.6 mm Phenomenex Luna Omega 5 μm PS C18 HPLC column. Samples were eluted with a gradient of acetonitrile and water with 0.1% TFA at a flow rate of 1.5 mL/min. 20% acetonitrile in water was used from 0 to 5 min, and a gradient of 20% to 50% acetonitrile in water was used from 5 to 25 min.

HPLC Method B: HPLC chromatograms were acquired using a Shimadzu 40 Series HPLC system equipped with a radio-detector and a 150 × 4.6 mm Phenomenex Jupiter Proteo C18 HPLC column. Samples were eluted with a gradient of acetonitrile and water with 0.1% TFA at a flow rate of 2.0 mL/min. 10% acetonitrile in water was used from 0 to 2 min, and a gradient of 10% to 50% acetonitrile in water was used from 2 to 15 min.

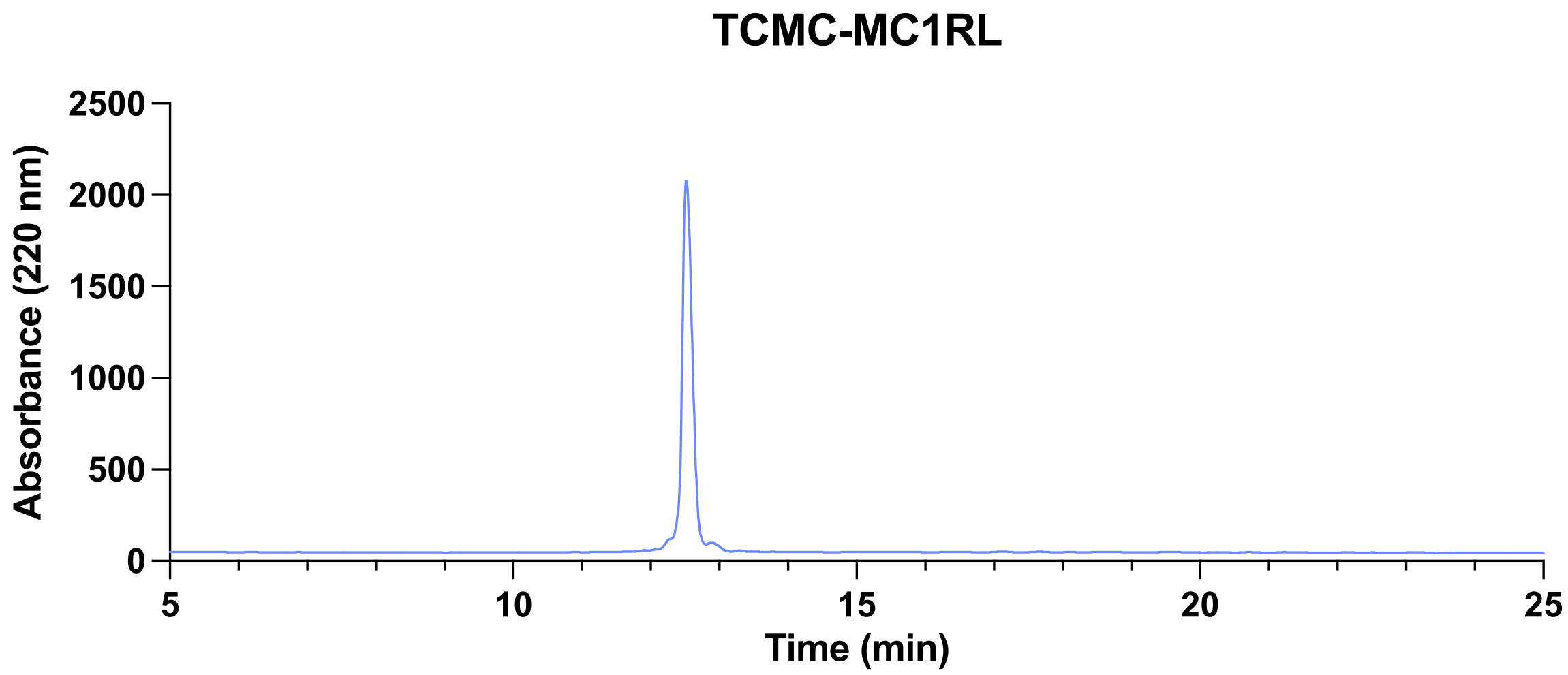

Figure S8: HPLC trace of TCMC-MC1RL (HPLC Method A).

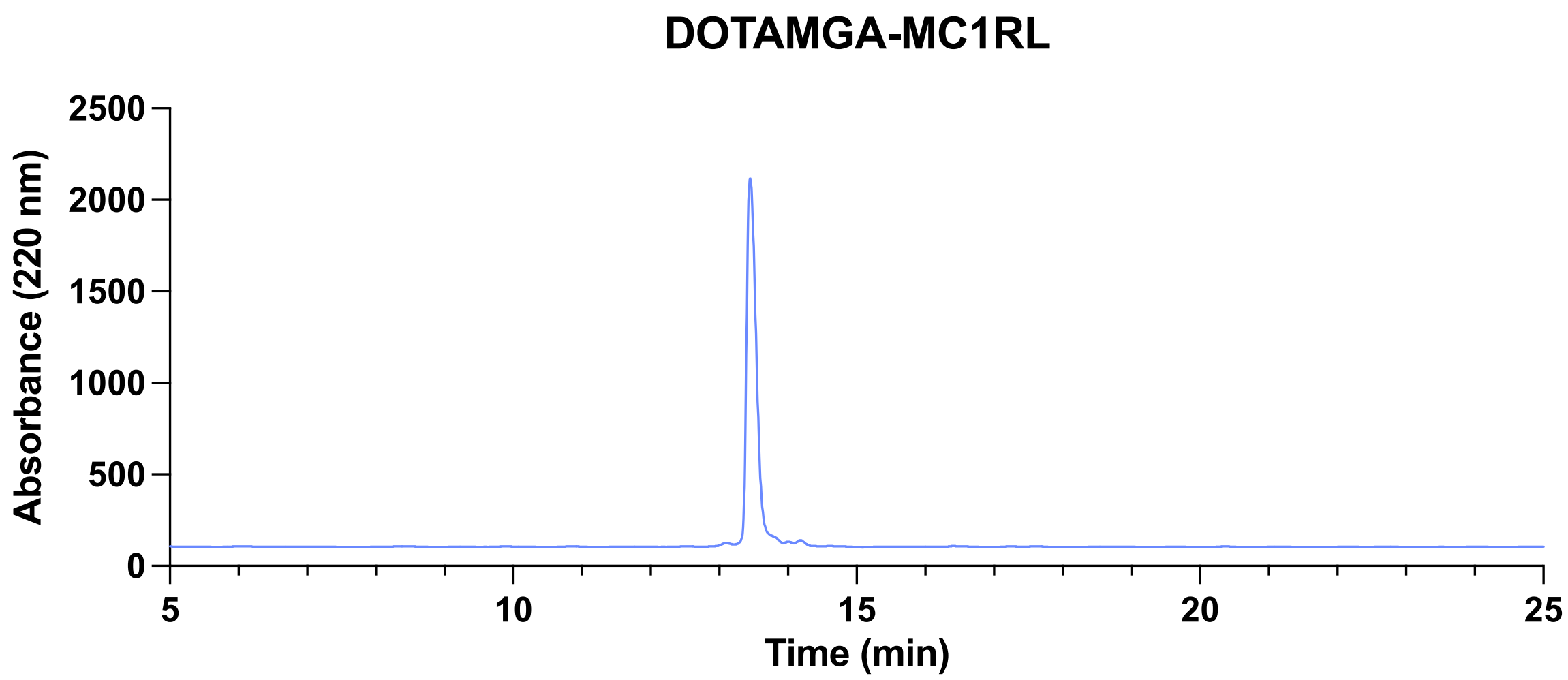

Figure S9: HPLC trace of DOTAMGA-MC1RL (HPLC Method A).

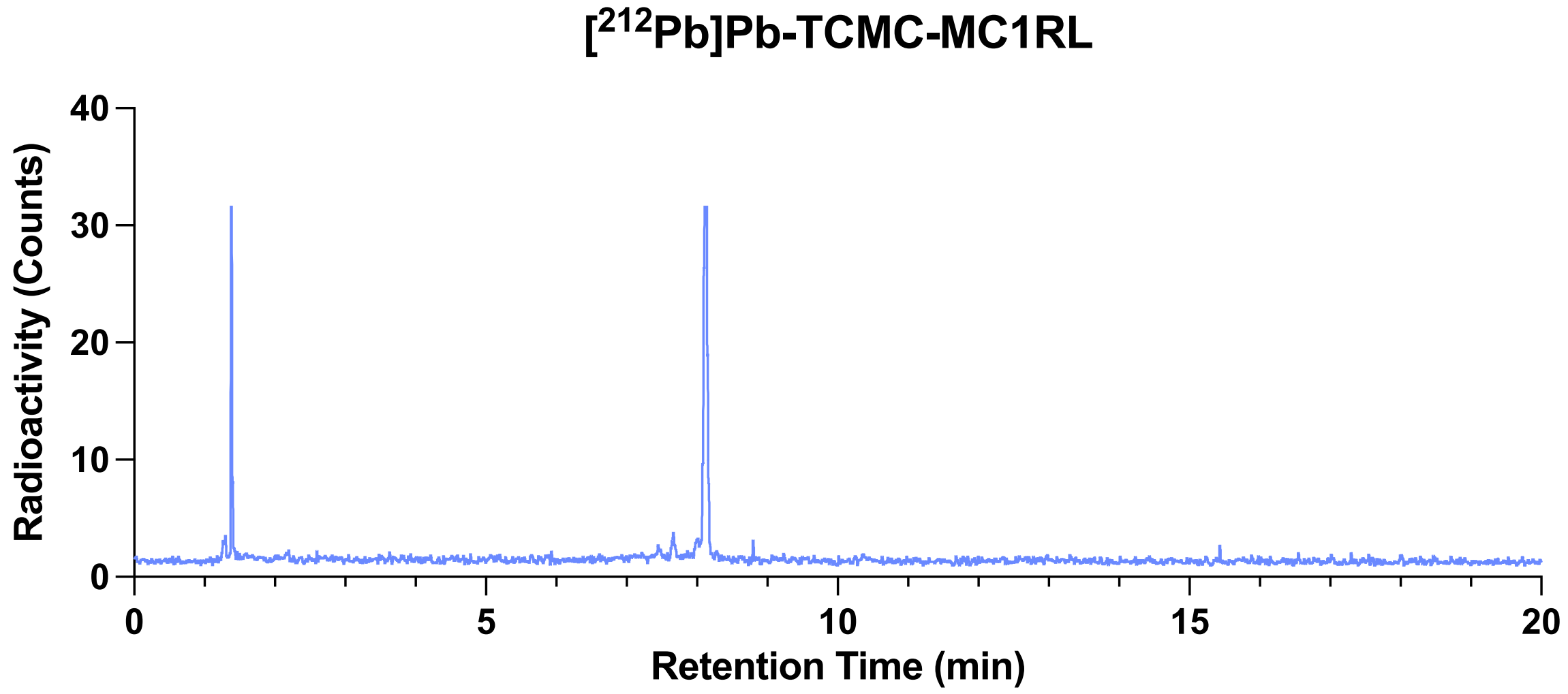

Figure S10: Radio-HPLC trace of [^{212}Pb]Pb-TCMC-MC1RL (HPLC Method B).

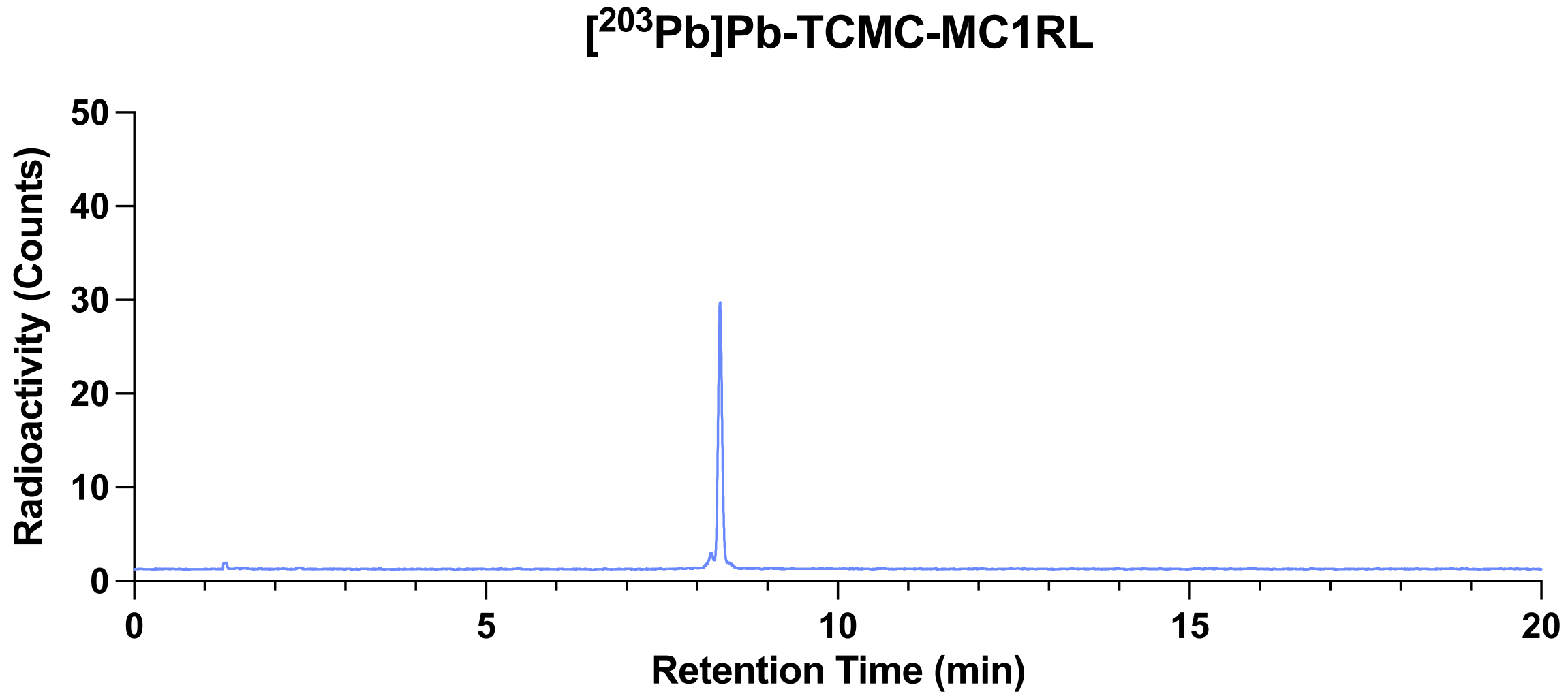

Figure S11: Radio-HPLC trace of [^{203}Pb]Pb-TCMC-MC1RL (HPLC Method B).

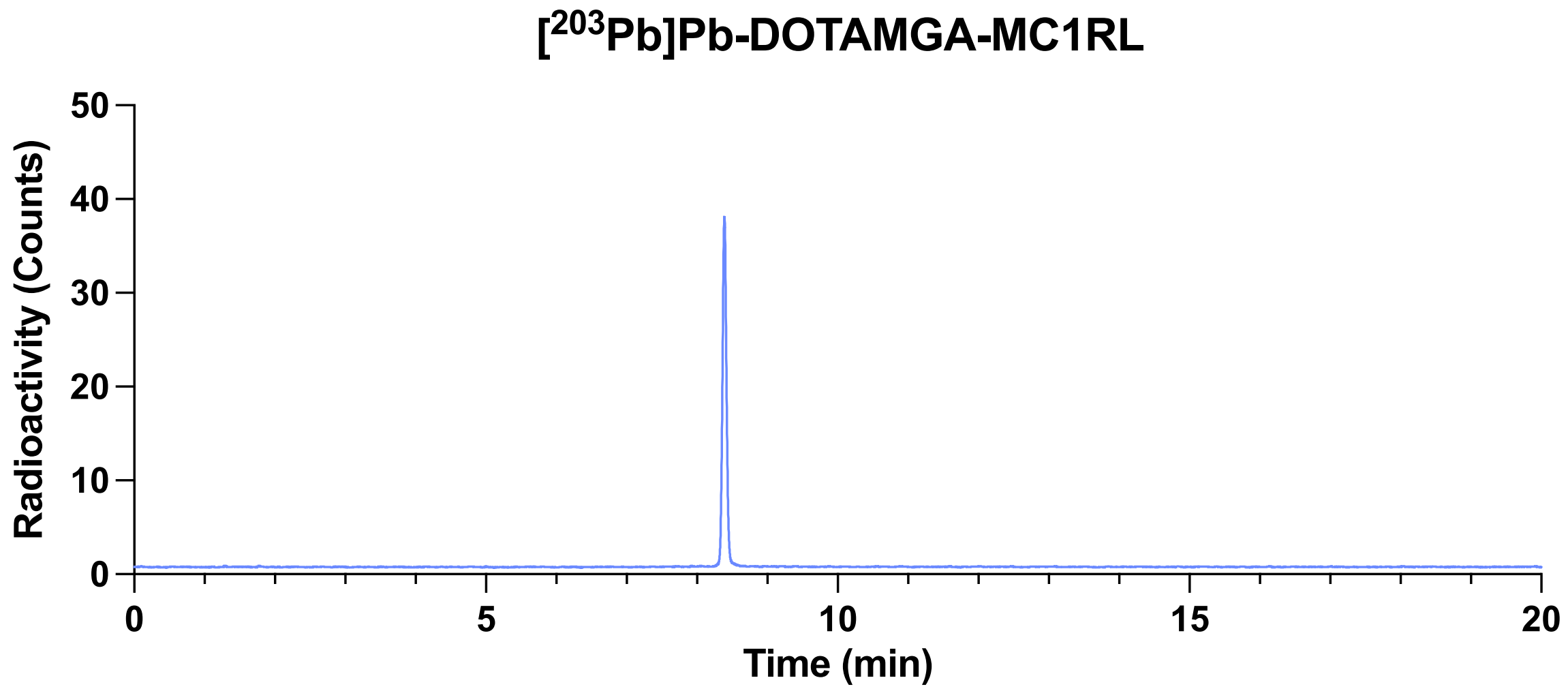

Figure S12: Radio-HPLC trace of [^{203}Pb]Pb-DOTAMGA-MC1RL (HPLC Method B).

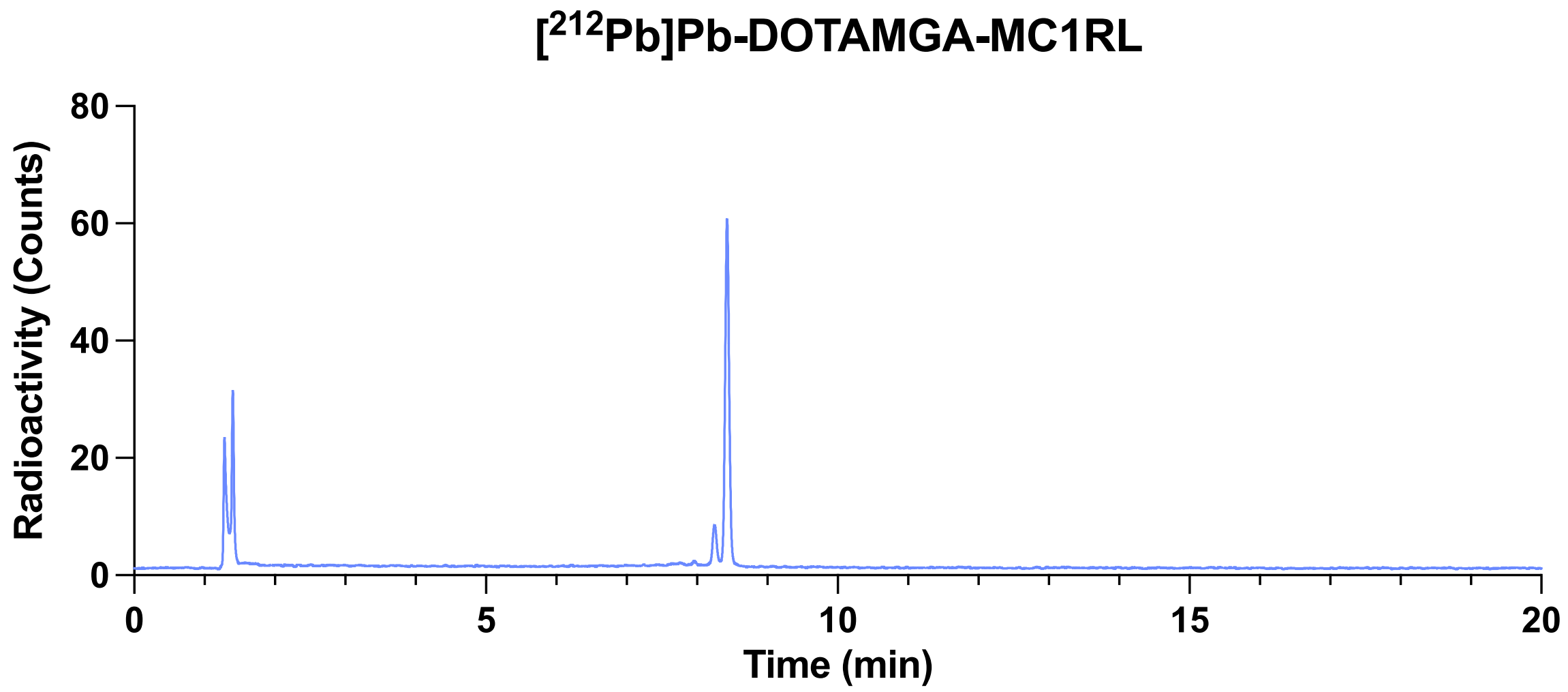

Figure S13: Radio-HPLC trace of [^{212}Pb]Pb-DOTAMGA-MC1RL (HPLC Method B).

S4. Peptide Characterisation & Purity

Table S1: Purity and characterisation data of unlabelled peptides.

Compound	Purity [a]	t_R [b]	HRMS m/z
TCMC-MC1RL	97%	12.17 min	$[M + 2H]^{2+}$ for $C_{72}H_{107}N_{22}O_{12}^{2+}$: calcd. 736.4253; found 736.4221
DOTAMGA-MC1RL	96%	13.45 min	$[M + 2H]^{2+}$ for $C_{75}H_{111}N_{22}O_{14}^{2+}$: calcd. 772.4359; found 772.4359

[a] Purity determined by integrating HPLC peak areas. [b] HPLC Method A.

Table S2: Radiochemical purity and radiochemical conversion of ^{203}Pb radiolabelled peptides for the *in vivo* studies.

Compound [a]	RCP [b]	RCC [c]
[^{203}Pb]Pb-TCMC-MC1RL [d]	89%	94%
[^{203}Pb]Pb-DOTAMGA-MC1RL [e]	> 99%	> 99%

[a] Radiolabelling performed in pH 5.5 NaOAc. [b] Determined by integrating radio-HPLC peak areas. [c] Determined by radio-TLC of the reaction mixture. [d] Radiolabelling reaction heated at 50 °C for 50 min. [e] Radiolabelling reaction heated at 50 °C for 20 min.

S5. *Ex Vivo* Biodistribution

Table S3: *Ex vivo* biodistribution of [^{203}Pb]Pb-TCMC-MC1RL in female B16F0 bearing C57BL/6 mice. Results reported as mean ± standard deviation (*n* = 3 unless otherwise stated).

**Organ**	**1 h [a]**	**3 h**	**24 h**
Bladder	0.83 ± 0.022	0.76 ± 0.89	0.13 ± 0.083
Blood	0.39 ± 0.042	0.053 ± 0.012	0.037 ± 0.012
Brain	0.022 ± 0.003	0.0073 ± 0.001	0.0052 ± 0.007
GIT	0.16 ± 0.009	0.15 ± 0.015	0.088 ± 0.050
Heart	0.14 ± 0.005	0.035 ± 0.006	0.026 ± 0.012
Kidneys	5.2 ± 0.54	4.6 ± 0.54	2.9 ± 0.16
Liver	15 ± 0.22	14 ± 2.3	12 ± 2.0
Lungs	0.43 ± 0.006	0.13 ± 0.026	0.083 ± 0.032
Skin	0.30 ± 0.002	0.055 ± 0.002	0.020 ± 0.018
Spleen	2.9 ± 1.4	2.2 ± 0.56	2.0 ± 0.32
Tumour	1.4 ± 0.37	0.74 ± 0.018	0.40 ± 0.050

[a] *n* = 2

Table S4: *Ex vivo* biodistribution of [^{203}Pb]Pb-DOTAMGA-MC1RL in female B16F0 bearing C57BL/6 mice. Results reported as mean ± standard deviation (*n* = 3).

**Organ**	**1 h**	**3 h**	**24 h**
Bladder	0.68 ± 0.36	0.46 ± 0.067	0.010 ± 0.014
Blood	0.89 ± 0.23	0.20 ± 0.007	0.14 ± 0.023
Brain	0.037 ± 0.015	0.011 ± 0.001	0.0071 ± 0.0002
GIT	0.30 ± 0.062	0.35 ± 0.006	0.11 ± 0.008
Heart	0.35 ± 0.045	0.084 ± 0.016	0.06 ± 0.010
Kidneys	5.2 ± 0.81	4.2 ± 0.22	3.3 ± 0.081
Liver	21 ± 2.9	25 ± 5 0.73	15 ± 5 2.6
Lungs	1.0 ± 0.17	0.39 ± 0.003	0.13 ± 0.066
Skin	0.58 ± 0.083	0.11 ± 0.012	0.062 ± 0.027
Spleen	3.5 ± 1.4	3.0 ± 0.44	2.7 ± 0.22
Tumour	3.9 ± 0.87	3.7 ± 1.3	1.4 ± 0.055
